# Cerebral *Corpora amylacea* are dense membranous labyrinths containing structurally preserved cell organelles

**DOI:** 10.1038/s41598-018-36223-4

**Published:** 2018-12-21

**Authors:** Paula P. Navarro, Christel Genoud, Daniel Castaño-Díez, Alexandra Graff-Meyer, Amanda J. Lewis, Yvonne de Gier, Matthias E. Lauer, Markus Britschgi, Bernd Bohrmann, Stephan Frank, Jürgen Hench, Gabriel Schweighauser, Annemieke J. M. Rozemuller, Wilma D. J. van de Berg, Henning Stahlberg, Sarah H. Shahmoradian

**Affiliations:** 1 Center for Cellular Imaging and NanoAnalytics (C-CINA), Biozentrum, University of Basel, Basel, Switzerland; 2 Friedrich Miescher Institute for Biomedical Research, Basel, Switzerland; 3 Amsterdam UMC, Vrije Universiteit Amsterdam, Department of Anatomy and Neurosciences, Section Clinical Neuroanatomy and Biobanking, Amsterdam Neuroscience, De Boelelaan 1117, Amsterdam, The Netherlands; 4 Roche Pharma Research and Early Development, Chemical Biology, Roche Innovation Center Basel, Basel, Switzerland; 5 Roche Pharma Research and Early Development, Neuroscience, Ophthalmology, and Rare Disease Discovery and Translational Area/Neuroscience Discovery, Roche Innovation Center Basel, Basel, Switzerland; 6 Division of Neuropathology, Institute for Medical Genetics and Pathology, University Hospital Basel, Basel, Switzerland; 7 Amsterdam UMC, Vrije Universiteit Amsterdam, Department of Pathology, Amsterdam Neuroscience, De Boelelaan 1117, Amsterdam, The Netherlands; 8 Department of Biology and Chemistry, Paul Scherrer Institute, Villigen, Switzerland; 9 BioEM Lab, Center for Cellular Imaging and NanoAnalytics (C-CINA), Biozentrum, Universtiy of Basel, Basel, Switzerland

## Abstract

*Corpora amylacea* are cell-derived structures that appear physiologically in the aged human brain. While their histological identification is straightforward, their ultrastructural composition and microenvironment at the nanoscale have remained unclear so far, as has their relevance to aging and certain disease states that involve the sequestration of toxic cellular metabolites. Here, we apply correlative serial block-face scanning electron microscopy and transmission electron tomography to gain three-dimensional insight into the ultrastructure and surrounding microenvironment of cerebral *Corpora amylacea* in the human brainstem and hippocampal region. We find that cerebral *Corpora amylacea* are composed of dense labyrinth-like sheets of lipid membranes, contain vesicles as well as morphologically preserved mitochondria, and are in close proximity to blood vessels and the glymphatic system, primarily within the cytoplasm of perivascular glial cells. Our results clarify the nature of cerebral *Corpora amylacea* and provide first hints on how they may arise and develop in the aging brain.

## Introduction

*Corpora amylacea* (CA) are hyaline glycoprotein-rich aggregates^1,2^ that are observed with increasing frequency in the aging human brain, particularly in the ventricular and subventricular regions^3–5^. Since their initial discovery in 1837 by Purkinje^6^, the biological role of CA in aging, their origin, mechanism of formation and ultrastructure remain unclear^7^. The name *Corpora amylacea* derived from the Latin ‘corpus’, body; ‘amylaceous’, starchy, refers to the amyloid polysaccharide composition historically demonstrated by positive Periodic Acid Schiff (PAS) histological staining and light microscopy (LM), and by ultrastructural studies that revealed structures originally interpreted as amyloid-like fibrils. Glycogen is the storage form of polyglucosan in eukaryotic cells. In brain tissue, glycogen has primarily been identified within astrocytes^8,9^, although research has recently shown that neurons possess an active glycogen metabolism that can lead to the intraneuronal accumulation of this polysaccharide^10^. The aggregation and/or accumulation of glycogen is directly correlated to aging and disease^11^.

CA are found in the physiological aging brain and are distinct from the clinically relevant aggregates of abnormal glycogen collectively known as polyglucosan bodies (PGB), which primarily differ from one another in terms of the medical symptoms associated with their occurrence^12^. PGB include Lafora bodies, Bielschowsky bodies, and amylopectin bodies, among others. They are found in the central and peripheral nervous system and also in other organs. Cerebral CA are also distinct from ‘*Corpora amylacea’* found in the heart, liver, prostate, lung and thyroid. The composition of the latter aggregates also differs between organs; only those in the heart have the same polyglucosan content as CA found in the central nervous system (CNS)^3^.

CA of the CNS contain abnormally branched glycogen, specifically amylopectin, with small amounts of protein, sulphate and phosphate groups^13–15^. They are generally defined as dense bodies at the distal processes of astrocytes that lack a limiting or enclosing membrane and, therefore commonly acknowledged as extracellular aggregates rather than typical inclusion bodies^16–18^. However, an intracellular origin of CA in glial cells as a result of phagocytosis of residual metabolites followed by glial degeneration, has also been postulated^12^. Moreover, some studies have indicated CA location within axons, suggesting a neuronal origin^19,20^. In CNS tissue, CA are thought to originate in neurons or astroglia, but also theories on CA being debris of brain cells from astroglial origin are strongly suggested by literature^3,12,16^.

Based on two-dimensional (2D) EM, the CA ultrastructure in the human brain has been described as being composed of tightly aggregated fibrils^21^ and as tangles of short linear fibrils with a variable diameter (8–12 nm)^12,16^. The diameter of the fibrils reported to form the ultrastructure of ‘*Corpora amylacea’* found in other organs ranges from 6–7 nm in the heart^22^, 7–10 nm in the liver^23^ to 12.5 nm in the prostate^24^. According to Leel-Ôssy, cerebral CA have an inner core that is 50–100 nm in diameter and the fibrils present in the tangle are the same as fibrils found in astrocytes (glial fibrillary acidic protein (GFAP))^18^. Additional ultrastructural studies on the CNS of aged cats and dogs describe the ultrastructure of CA as apparently randomly branching fibrils, 8–9 nm in diameter, that are associated with glycogen granules^25,26^.

The formation of CA, and some PGB, in the CNS is possibly related to deregulation of storage pathways that involve glycogen^3^. Indeed, studies using genetically modified mice and tissue cultures have shown that upregulation of glycogen synthesis provokes neuronal dysfunction^10^, which manifests itself as protein aggregates, and is linked to the occurrence of age-dependent amassments of diverse aggregation-prone proteins and oxidative stress^11^. LM suggests that CA in the brain display epitopes for many antibodies, which has led to ambiguous descriptions of their protein content^1,2,27–30^. Importantly, hsp70 and ubiquitin are present, and also alpha-synuclein (aSyn)^31^ albeit to a lesser extent than in Lewy bodies (LB)^32^. LB, the hallmark of Parkinson’s disease (PD), are intraneuronal globular aggregates that resemble CA in size, but show a very high concentration of aSyn by immunohistochemical analysis, and, unlike CA, are not stained positively by PAS. CA have been identified in Alzheimer’s disease (AD), amyotrophic lateral sclerosis (ALS) and PD patients, with similarity in anatomic location, morphology and epitopes.

Several microscopically apparent inclusions and deposit-body-like structures can be found in aged and diseased human brain tissue. In addition to CA and PGB, others include Psammoma, Negri, Pick, Bunina, Hirano, and Zebra bodies as well as ubiquitin-positive inclusions associated with motor neuron disease^12,33,34^ and several types of extracellular plaques^35^. While most of these aggregates have been broadly studied, the lack of high-resolution and high-quality information about their ultrastructure makes difficult the possibility to clearly define their composition and origins. The rarity of well-preserved post-mortem human brain tissues from aged donors that are suitable for electron microscopy (EM) is one of the main factors underlying this scarcity of high-resolution ultrastructural data.

In the work reported here, we utilized advanced 3D EM approaches to resolve the ultrastructure of CA found within the hippocampal CA2 sector and brainstem *Substantia nigra pars compacta* (SNpc) and *canalis centralis* of post-mortem tissue from six donors aged 76 to 92 years, three of them diagnosed as having suffered from PD pathology. The tissue was prepared using rapid-autopsy and improved sample preparation protocols to ensure high ultrastructural preservation. We combined LM with 3D EM approaches, including correlative SEM/TEM characterization of CA at the nanometer level. Such ultrastructural studies are essential to understand the biological origin of cerebral CA and their relevance to aging and certain disease states that involve the sequestration of toxic cellular metabolites^36–38^.

## Results

The detailed information of all donors as well as an overview of the data shown in this study is summarized in Supplementary Table S1.

### Localization and characterization of *Corpora amylacea*

**Figure 1:**
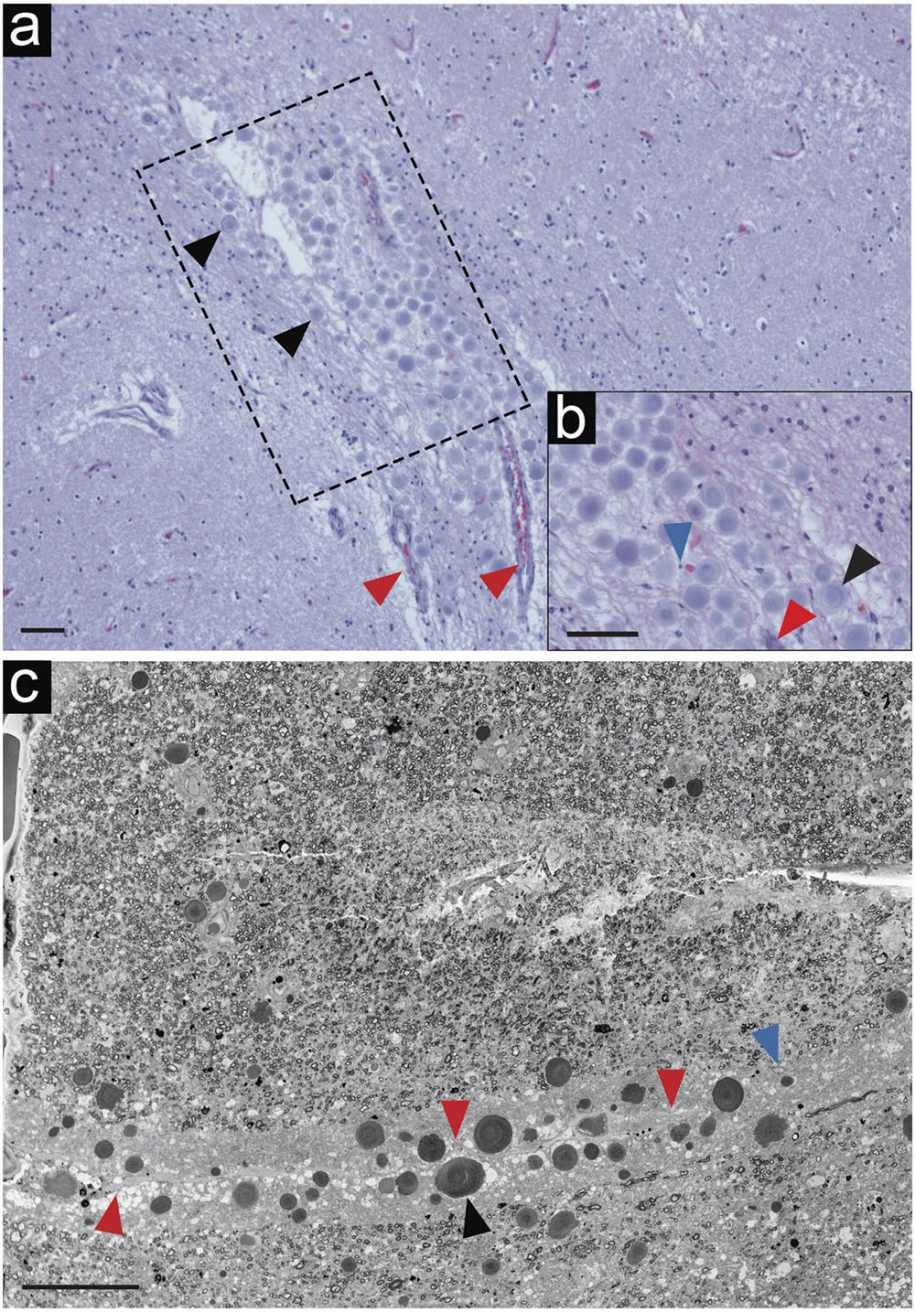
Localization of *Corpora amylacea* in human brain, as visualized by light and electron microscopy. Human brain tissue from donor B, diagnosed with PD. (**a**) H&E staining revealing CA in the hippocampus region adjacent to the lateral ventricle. (**b**) High magnification image of (**a**) region where some cell nuclei and the vascular system of the brain are in close proximity to CA. (**c**) SBF-SEM image showing CA within a directly adjacent tissue section. The region shown corresponds to the black box (dotted lines) in (**a**). The tomogram including this region is shown in Supplementary Movie 1. Black arrowheads = CA; red arrowheads = blood vessels; blue arrowheads = nuclei. Scale bars = 60 µm.

Haematoxylin and eosin (H&E) and periodic acid Schiff (PAS) staining were used to identify CA within hippocampus and SNpc from donors. By this classical LM analysis, CA appeared primarily as extracellular purple/pink-colored aggregates, displaying the characteristic concentric ring staining pattern and overall morphology (Fig. 1a,b, black arrowheads). In addition, many pink dots (PAS-positive) appeared to fill the cytoplasm and were in close proximity to cell nuclei (Supplementary Fig. S1b,d,f,h,l, black arrowheads). These smaller, apparently intracellular CA were often found in the proximity of vascular structures and frequently near the surface of the ventricular and subventricular brain regions (Fig. 1a,b and Supplementary Fig. S1, red arrowheads). Location and distribution of CA were found to be homogenous amongst all donors, appearing prominently at the hippocampal fold, dentate gyrus (DG) and hilus (Fig. 1, Supplementary Figs S1 and S2a,b, black arrowheads). CA were most abundant in hippocampus from donors A, B and D, and in the SNpc from donors B and C. Overall, the hippocampus presented a significantly higher abundance of CA as compared to brainstem amongst donors by unpaired t-test statistical analysis (Supplementary Fig. S2d). EM data was acquired in areas of sections that were identified by LM to contain abundant CA.

Once the presence and distribution of CA had been confirmed in a human brain sample, adjacent 60 μm-thick tissue sections were cut and analyzed by LM, and prepared for investigation by EM. We used serial block face scanning electron microscopy (SBF-SEM) to localize CA in the post-mortem human brain samples followed by correlative higher-resolution TEM to image their ultrastructure.

Whole tissue block (~1 mm × 1 mm × 60 μm) SBF-SEM imaging of heavy metal-stained, resin-embedded hippocampal tissue from donor B showed CA as spherical, electron dense aggregates located next to brain blood vessels (Fig. 1c, Supplementary Fig. S3a, and Supplementary Movie 1). SBF-SEM images show that in brain tissue CA are surrounded by axons, cell nuclei, and lipofuscin amongst other intracellular and extracellular material and cell organelles.

**Figure 2:**
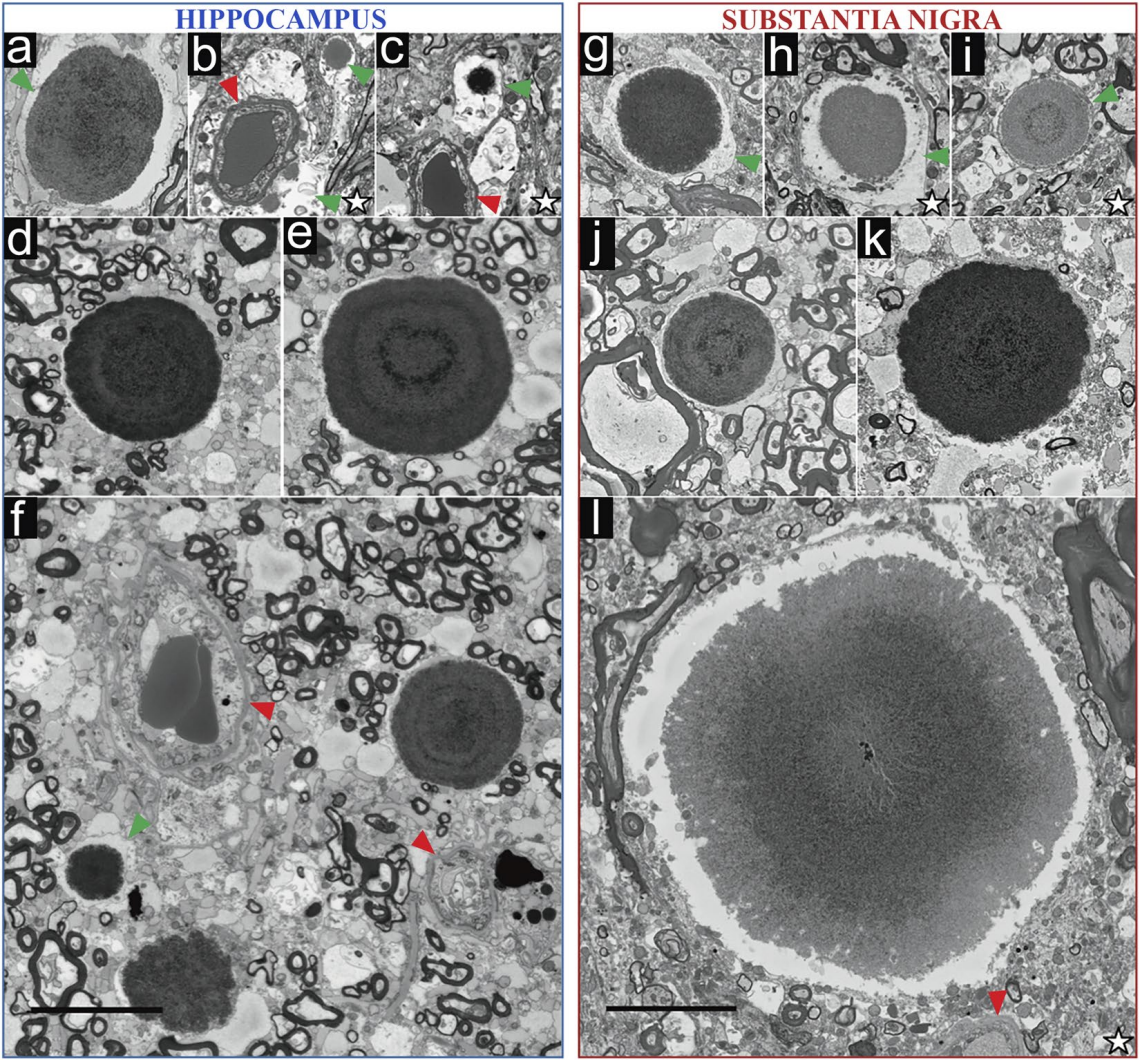
Morphology of heavy metal-stained *Corpora amylacea*, as visualized by SBF-SEM. Each image represents a single 2D slice from a 3D stack recorded from CA identified in hippocampus or SNpc of donors A, B, C and E (Supplementary Table 1). The electron micrographs were recorded from (**a**) CA2 hippocampus of donor A, (**d**–**f**) CA2 hippocampus of donors B, (**b,c**) CA2 hippocampus of donor C, (**g**,**j**,**k**), SNpc of donor B, (**h**,**i**,**l**) and C. Green arrowheads = cell cytoplasm containing CA, thereby considered as intracellular; red arrowheads = blood vessels; white star = non-demented aged donors. Non-starred images represent images from PD donors. All panels are reproduced at the same scale. Scale bar = 10 µm.

To further characterize cerebral CA, we collected additional higher resolution SBF-SEM data from stained hippocampus, SNpc and *canalis centralis* regions of post-mortem brain tissue from three additional donors. The morphology of the CA imaged are summarized in Fig. 2 and extended in Supplementary Fig. S4. In these data sets (16–8 nm/pixel), smaller CA (0.5–5 μm diameter) and their surroundings were more clearly visualized than by LM, providing a 3D view of CA embedded in human brain tissue. This higher resolution data also clearly show that some smaller CA are enclosed by a membrane and are thereby intracellular (Fig. 2, Supplementary Figs S3 and S4 and Supplementary Movies 1 and 2). In all cases, CA were visualized as electron-dense aggregates with a granular appearance often close to blood vessels, and were composed of layers of concentric rings mimicking a bull’s eye (Figs 1, 2 and Supplementary Figs S1–4). All ~160 image stacks of partially and completely imaged CA (a total of 335 CA) found in the 60 μm-thick brain sections were reconstructed in 3D. Supplementary Fig. S5 and Movies 1–3 show typical examples and document the spherical to ellipsoid shape of CA. The reconstructed volumes extend over a tissue thickness of 10–45 μm per CA. In Supplementary Movies 2A, 3D color segmentation is employed to illustrate the layered staining pattern typical of CA. A higher number of concentric rings were found in larger CA (Figs 1c, 2, Supplementary Figs S3, S4 and Supplementary Movies 1 and 2). In this study, no difference between CA in human brains from non-demented donors and from PD donors was observed by both LM and SBF-SEM. CA from both types of donors shared the same location, distribution and morphological features. However, the abundance of CA differed amongst donors (Supplementary Figs S1 and S2).

Based on our SBF-SEM data, we conclude that CA are composed of concentric layers of compacted material of varying composition, which results in the distinctive staining pattern. Consequently, we suggest that components of various cellular origins (cell organelles, lipid-rich structures as lipofuscin and vesicles) join the bulk of the CA structure (Supplementary Fig. S3 and Movie 2B).

### Differences between intracellular and extracellular *Corpora amylacea* and their distribution relative to the vascular system

According to our image data, intracellular CA are typically smaller than 10 µm in diameter, and appear less abundant than extracellular CA. They have the same texture as extracellular CA and display the same concentric ring pattern, but show fewer rings that could be mainly attributable to their small size; furthermore, they also localize within the brain vasculature (Fig. 2 (green arrowheads), Supplementary Figs S1 and S4 (green asterisk), S5, S6 and Supplementary Movies 2 and 3). In contrast to extracellular CA, intracellular CA are enclosed by the cytoplasmic membrane of a cell. Moreover, extracellular CA are larger aggregates up to 30 µm in diameter, surrounded by myelin sheaths and cells. A quantitative comparison between intracellular and extracellular CA morphology is summarized in Supplementary Fig. S5.

**Figure 3:**
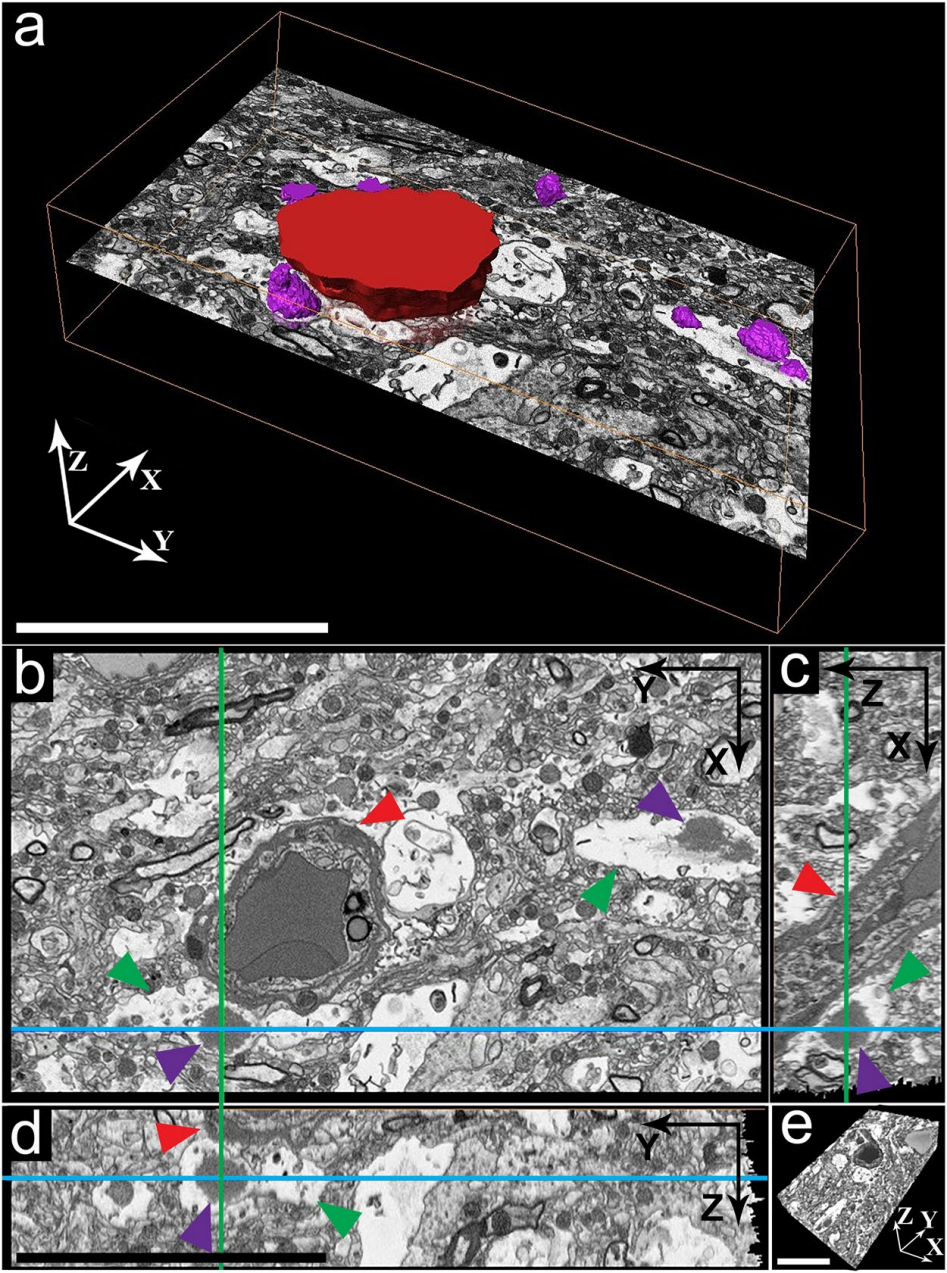
3D morphology and localization of *Corpora amylacea* in hippocampus of non-demented aged donor C, as visualized by SBF-SEM. (**a**) 3D surface reconstruction of the SBF-SEM data stack showing CA (purple surfaces) and a blood vessel (red surface). (**b**–**d**) 2D orthoslices of SBF-SEM data from CA found in (**a**). The slice axes are indicated by blue and green lines and were set at one of the CA. (**b**) XY orthoslice, (**c**) XZ orthoslice and (**d**) YZ orthoslice. (**e**) The whole reconstructed 3D volume. Red arrowheads = blood vessels (including basal lamina), purple arrowheads = CA and green arrowheads = astroglial feet forming the neurovascular unit of the vascular system of the brain. Scale bars = 10 µm.

Cell organelles are found in the bulk of CA and their immediate surroundings in both intracellular and extracellular CA. Furthermore, our 3D EM data reveal these organelles to be sometimes directly interacting with their edges and appearing to become incorporated into the bulk of the aggregate (Fig. 2, Supplementary Figs S3, S4, S6 and Supplementary Movies 2 and 3). To further investigate the relationship between intra- and extracellular CA and blood vessels, we used 3D-color segmentation to highlight these features as 3D-color rendered surfaces in five aligned SBF-SEM stacks (Supplementary Movie 3). The neurovascular unit (NVU) and its cellular components are visualized (blood cells, endothelial cells, pericytes, basal lamina and astroglia endfeet)^39,40^ in 2D SBF-SEM images of individual specimen sections and 3D data (Fig. 3, Supplementary Fig. S3, Movies 2 and 3). Astroglial feet are identified in EM images by their typical location surrounding the basal lamina, and by the gap junctions they form with one another^40,41^. Glial processes displaying these features are easily identified in Fig. 3, Supplementary Fig. S7 and Supplementary Movie 3. Thus, according to our SBF-SEM images and tomograms, intracellular CA are found inside the cytoplasm of perivascular glial cells, specifically within endfoot processes that directly contact the surface of a blood vessel (Figs 2, 3, Supplementary Figs S6, S7 and Movie 3). Furthermore, in correlation to the PAS staining, several focal spots of CA aggregation can be found within a single glial process that is attached to a blood vessel and basal lamina containing pericytes (Fig. 3b–d, Supplementary Fig. S1 and Movie 3). This may indicate a mechanistic relationship between the brain’s vasculature and CA formation.

Two cuboid 60 μm-thick tissue pieces, in which we located several CA in close proximity to blood vessels, are shown in Fig. 3a,e and Supplementary Fig. S7a,b. Selected ROIs within these 3D volumes are visualized as XY, XZ and YZ 2D-planes (Fig. 3b–d), represented by slicing axes indicated by green and blue lines. The XZ and YZ planes (Fig. 3c,d, respectively) of the tissue from donor C hippocampus show a CA (purple arrowhead) surrounded by the cytoplasmic membrane of a glia cell (green arrowhead) attached to a blood vessel visualized in cross-section (Fig. 3b–d, red arrowhead). Several small intracellular CA are apparent in the corresponding XY plane (Fig. 3b, purple arrowheads and Supplementary Movie 3, purple surface). Two extracellular and one smaller intracellular CA were present in the tissue from donor B hippocampus, being only partially imaged due to sample drift (Supplementary Fig. S7). An extracellular CA is visible in all the three planes displayed for the reconstructed cuboid, and is situated next to a branched blood vessel (Supplementary Fig. S7). A pericyte (Supplementary Fig. S7c,e, yellow arrowhead) is evident between the CA (purple arrowheads) and the blood vessel (red arrowheads). All of our 3D surface reconstructions show that intracellular CA localize within the cytoplasm of glial cells embracing blood vessels, whereas extracellular CA localize in the vicinity of blood vessels and are not enclosed by an intact host-cell membrane (Fig. 3 and Supplementary Fig. S7). Interestingly, we have not visualized CA within neurons.

**Figure 4:**
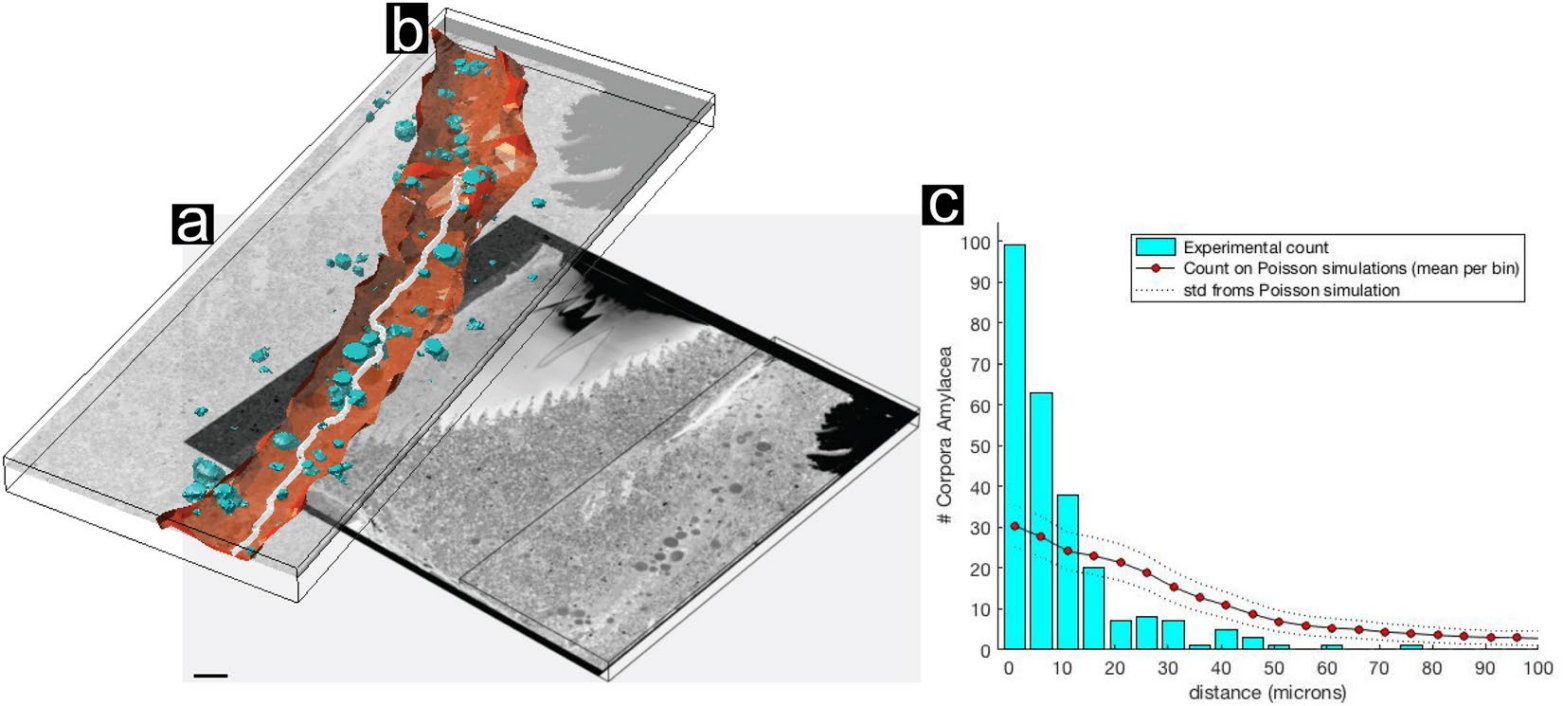
3D volume reconstruction of *Corpora amylacea* reveals their close proximity to blood vessels. (**a**) Z-orthoslice from a SBF-SEM 3D stack imaging a whole block of post-mortem human brain tissue (donor B, CA2 hippocampus). The entire tomogram is shown in Supplementary Movie 1, and a 2D image from the tomogram is shown in Fig. 1c. (**b**) 3D volume reconstruction of (**a**) showing a blood vessel in white, the glymphatic area in red and CA in turquoise. (**c**) Histogram (blue) showing the number of CA (y-axis) at specific distances from blood vessels in µm (x-axis) and a simulation showing the distribution expected when the same 259 CA are positioned at randomized locations (red dots; the dotted lines indicate the variance of the 10,000 randomization sets examined; see Methods). Scale bar = 50 µm.

In addition, we observed that CA not only have a tendency to cluster around blood vessels, but also at the brain-specific glymphatic system that surrounds them. The main function of the glymphatic system is to efficiently clear waste from the CNS through perivascular tunnels formed by astroglial cells^42^. Characteristic features previously described in EM literature^39,43,44^ allowed us to identify this system in our data set as narrow electron lucent tunnels running in parallel to blood vessels (Fig. 1c, Supplementary Fig. S4 (red arrowheads) and Supplementary Movie 1). To statistically validate our visual impression of CA clustering, we performed a 3D volume reconstruction of three entire ~1 mm^2^ (in X and Y dimensions) tissue blocks imaged by SBF-SEM and traced all CA and blood vessels present as detailed in the Methods section. The majority (92.6%) of the 259 CA identified in the ~1 mm^2^ in XY by 60 μm-thick tissue block were situated inside the glymphatic area, very close to a large blood vessel (Fig. 4). According to the measurements, ~37% of the CA (95 CA) are within 5 μm, and ~77% (199 CA) are within 10 μm from the closest blood vessel (Fig. 4c; see Method section). By contrast, there would only be a 10% probability of observing CA (30 CA) within 10 μm from the closest blood vessel according to a Poisson distribution simulating randomly distributed CA (Fig. 4c). This comparison reveals that the actual occurrence of CA clustering in the proximity of vascular vessels is not a result of random CA scattering, and that there must exist an underlying physiological link.

### Ultrastructure of *Corpora amylacea*

**Figure 5:**
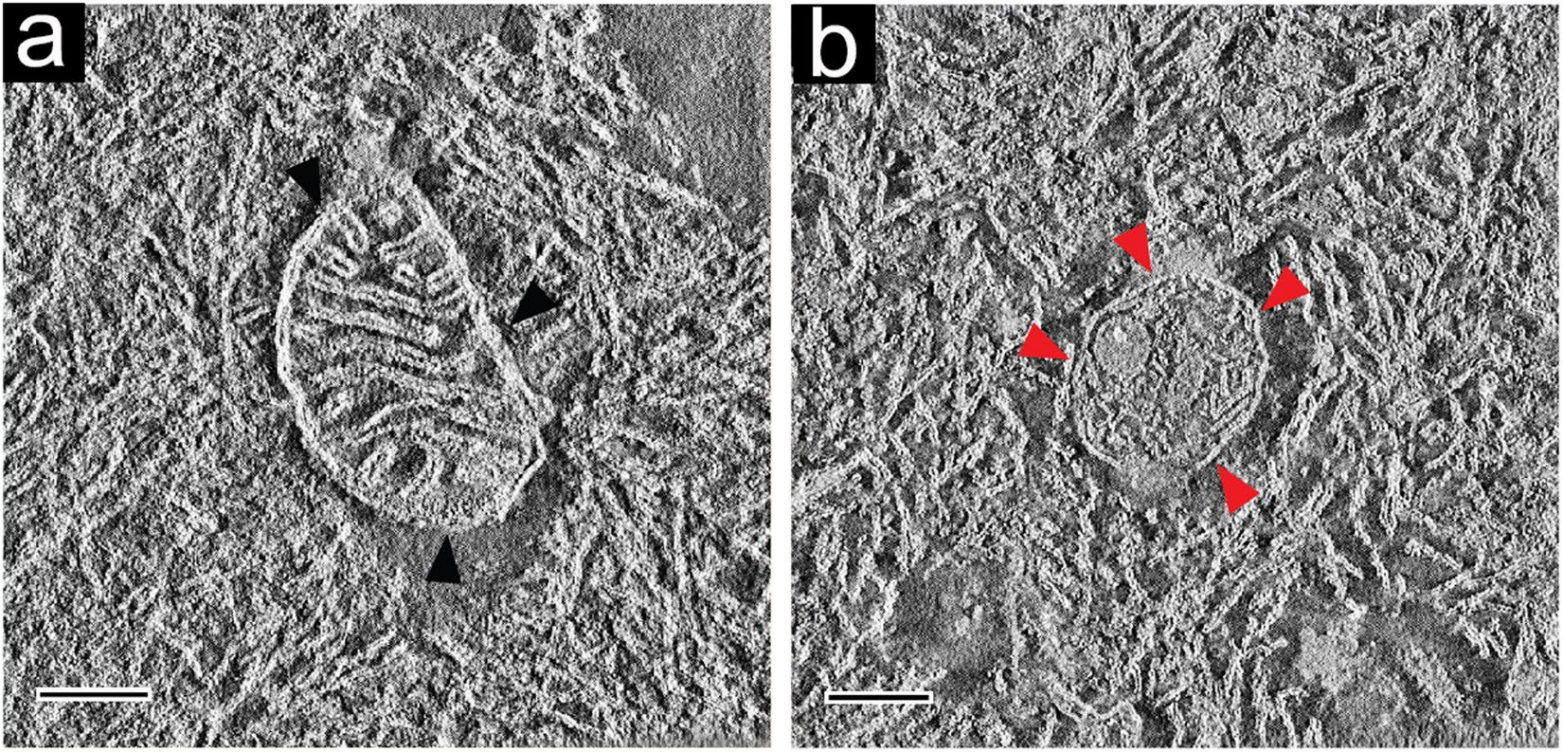
Ultrastructure of *Corpora amylacea* as revealed by TEM tomography. Anisotropic diffusion-filtered 2D images from reconstructed tomograms showing (**a**) the edge region of an extracellular CA; black arrowheads indicate a structurally preserved mitochondrion surrounded by fragmented biological membranes; (**b**) the inner area of a CA; red arrowheads indicate a preserved vesicle containing fragmented material, and a small vesicle. The vesicle is surrounded by fragmented biological membranes. Original non-filtered images are displayed in Supplementary Fig. S9c–e. The electron tomograms were recorded from the same CA shown in Fig. 2l and Supplementary Movie 2D. Data from brainstem SNpc of donor C. Scale bars = 200 nm.

We performed correlative SBF-SEM/TEM electron tomography (ET) in order to describe CA ultrastructure at higher resolution in 3D. Several electron tomograms were acquired and reconstructed for the CA (within SNpc of donor C) shown in Fig. 2l and Supplementary Movie 2D. The reconstructed ET projections and the anisotropically filtered tomograms of both CA reveal a convoluted labyrinth-like aggregate formed from what appears to be densely packed lipidic membrane fragments. The fragments appear similar to the plasma membranes of cells and to the membranes of cell organelles, e.g., mitochondria and vesicles (Fig. 5, Supplementary Fig. S8 and Supplementary Movies 4, 5). Indeed, whole mitochondria surrounded by membrane fragments were observed within this particular CA. The mitochondria displayed morphologically intact cristae^45^, but in such a state was not deemed functional (Fig. 5a). Furthermore, vesicles and less electron-dense material that we interpret as proteinaceous clumps were present within CA and along their surface (Fig. 5, Supplementary Fig. S9 and Supplementary Movies 2, 4). The vesicles themselves contained small vesicles and disrupted organellar structures together with membrane fragments. Furthermore, the vesicle in Fig. 5b showed signs of membrane disruption, with a leaking point at which proteinaceous clumps could be identified.

Semi-automated 3D segmentation and sub-tomogram analysis of the reconstructed tomograms revealed membranous structures forming CA and the continuity of the membranes throughout the resolved areas at the nanometer scale (Supplementary Figs S8, S9 and Supplementary Movies 4, 5). In addition, tomographic data on samples from the hippocampus of donor A reveals the edge of a CA and the cellular environment at a resolution and clarity never before shown, to the best of our knowledge (Supplementary Fig. S8). In the same tomogram, yet not within the CA itself, adjacent cytoplasm was shown to contain cytoskeletal filaments. These filaments served as a control for the 3D color segmentation and sub-tomogram averaging procedure; they were visible after 3D color segmentation and the averaged particles displayed the typical signature of a rod-like structure (Supplementary Fig. S8b.ii–d.ii). Analysis of the edge of the CA in the tomogram showed a lipid membrane pattern and the typical signature of a sheet-like structure after sub-tomogram averaging (Supplementary Fig. S8b.iii–d.iii). We did not detect any amyloid or fibrillar structures in the CA, even though our ET data could resolve filaments distal to them in cell cytoplasm, proving that our sample preparation and imaging strategy did not preclude the ability to resolve filamentous structures (Supplementary S8b.ii–d.ii and Supplementary Movie 5).

**Figure 6:**
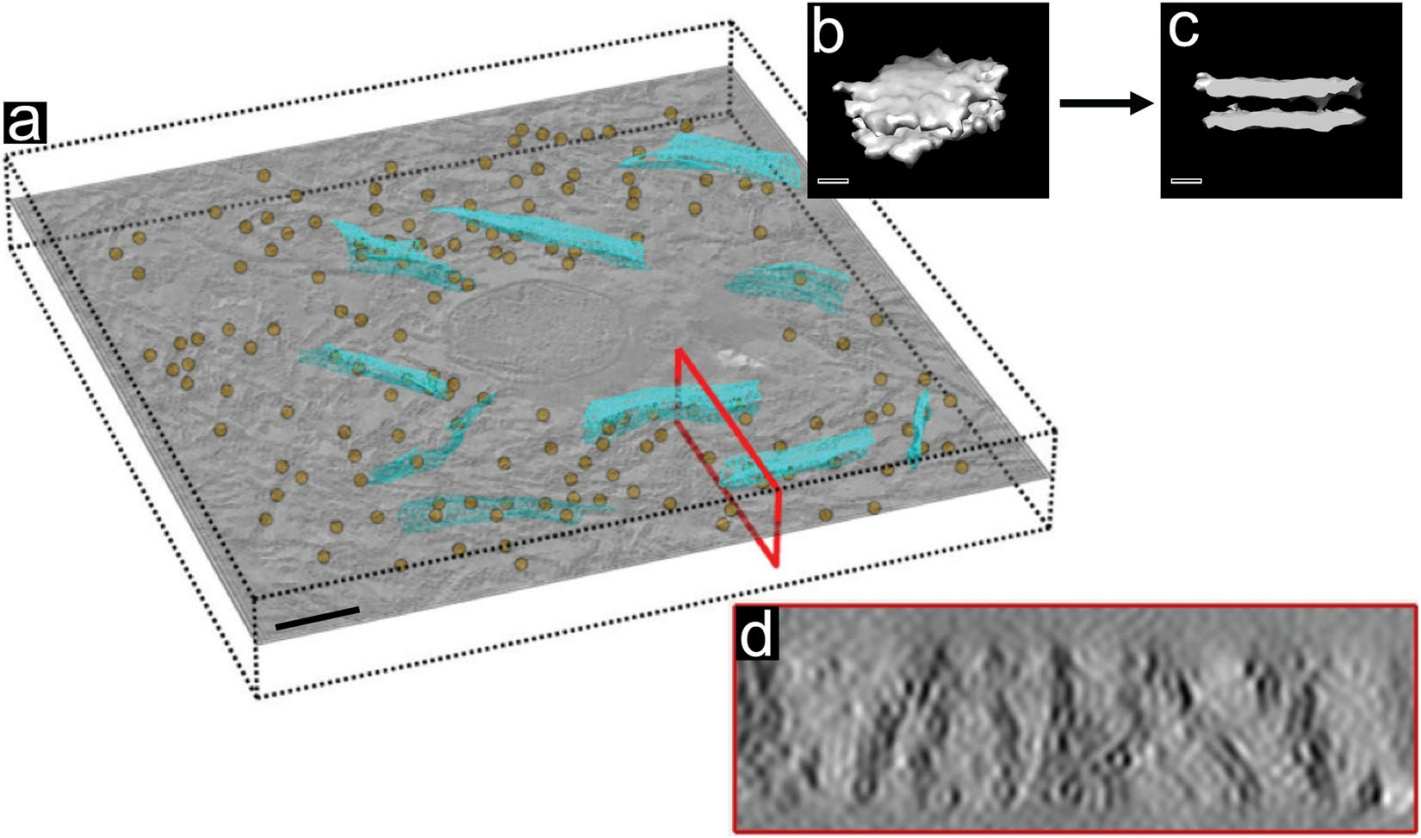
Sub-tomogram averaging of a TEM tomogram acquired of a *Corpora amylacea* reveals the presence of lipid membranes. (**a**) Z-orthoslice from a tomogram of CA, and YZ-orthoslice (red box). Brown dots indicate the structures selected within the tomogram for averaging. Blue meshes display the 3D triangulation for some of the selected structures. (**b**) Top view and (**c**) side view of the sampled structures after sub-tomogram averaging, revealing a membrane structure and the outer regions of the two lipid bilayer leaflets. (**d**) YZ-orthoslice of the tomogram showing the presence, continuity and torsion of the membranes throughout the tomogram. Data correspond to SNpc of donor C as shown in Supplementary Fig. S9f and Supplementary Movie 4. Scale bar = 200 nm.

TEM imaging showed that the average cross-sectional diameter of the membranous structures present in CA of the hippocampus and the SNpc were compatible with plasma membranes in the same tissue sections (Supplementary Table S2 and Fig. S6). The presence of coherent structural elements, such as membrane sheets, was explored by local texture analysis, whereby high contrast features were located within the CA tomograms and analyzed by sub-tomogram averaging. This revealed lipid bilayer membrane structures with a spacing of 6.9 nm ± 0.9 nm between the leaflets, measuring from inner surface to inner surface^46^ (Fig. 6 and Supplementary Fig. S8b.iii–d.iii, S9a.iii–f.iii). For the analysis, structures in the tomogram (Fig. 6a, brown dots) were reconstructed in 3D as a triangulated mesh in order to display continuity and torsion (Fig. 6a, blue triangulated mesh and Supplementary Fig. S10). Furthermore, averaged selected particles (more than 200 particles per tomogram) were reconstructed, revealing the surfaces of a membrane structure and the two leaflets of its bilayer (Fig. 6 and Supplementary Fig. S8). In addition, a cross section of the tomogram clearly shows membranes within the CA, revealing a lipid bilayer pattern as well as flexibility and torsion (Fig. 6 and Supplementary Fig. S10). The distances between the lipid bilayers visible in such slices were consistent with the membrane thickness of 6.9 nm measured by ET reported above, further supporting the conclusion that CA are composed of biological membrane fragments.

## Discussion

Here, we visualized CA applying cutting-edge EM techniques and in-depth 3D image analyses providing a solid basis for further research on the formation and ultrastructure of brain bodies. We describe CA morphology and location employing statistical quantification at the nanometer scale in highly preserved human brain tissue. Furthermore, a combination of SBF-SEM imaging and TEM tomography provides unprecedented high-resolution images of CA ultrastructure in their cellular context.

Based on high-resolution ultrastructure analysis by ET, we conclude that CA are primarily composed of densely packed aggregated lipid membrane fragments and disrupted cellular organelles (Fig. 5 and Supplementary Movies 3 and 4). We did not expect to find membranes, because previous studies employing histological staining methods and immunofluorescence labeling of CA epitopes did not detect lipids^14^. However, the CA examined in these were present in what we infer was paraffin sections of formalin-fixed tissue^11^. As lipids are soluble in the organic solvents employed during such sample embedding procedures, and, depending on their cellular context, are lost or highly depleted during the procedure^47^, we do not consider the absence of lipids in previous analyses to contradict our results. Importantly, the ultrastructural characterization of CA presented here goes beyond the information available from conventional 2D TEM imaging on which the current model is based. The two leaflets of a membrane are only distinguishable in a conventional 2D projection image when the membrane is precisely perpendicular to the imaged specimen plane. Any deviation from this makes the bilayer undetectable, or at least delivers an underestimate of its thickness (Supplementary Table 2). Such effects might explain why CA in the brain were thought to be comprised of polysaccharide fibrils^3,12,18^. Our 3D TEM data did not reveal any fibrils or amyloid-like structures within CA.

Literature data show that CA are immunoreactive for mitochondrial proteins^3,31,48–50^, complement cascade components^51^, glycation end-products^9,52–55^, microbial proteins^30,56^ and many proteins related to lipid metabolism and recycling^12,53,54,57,58^. It is well known that lipid-rich structures can lead to false positives in LM data when one attempts to localize specific proteins by immunofluorescence^59^. It also seems plausible that the aggregated nature of CA causes them to react immunohistochemically with a large number of antibodies. The possibility of false positives from the contamination of commercial antibodies with IgMs was also recently demonstrated^29^. In addition, new research on label-free multiphoton techniques have been able to identify CA in pathological human brain^55,60^. Thus, studies based on immunolabeling alone may not be the most appropriate tool to define CA composition and origin, and potentially many other kinds of cellular inclusions and deposit-body-like structures. In this study, we have demonstrated SBF-SEM and TEM tomography as valuable tools for revealing ultrastructural details that would otherwise not be possible to resolve with such clarity: specifically, we have visually identified mitochondria, organelles resembling lysosomes, digestive vesicles, glycogen granules and small organelle-like structures that compose the core and surface of CA (Supplementary Movies 2–5). Based on our 3D EM data, we suggest that CA originate in the glial feet that form part of the NVU and that their occurrence is tightly linked to the glymphatic system (Fig. 4 and Supplementary Movies 1–3). Although an intracellular location for CA have been suggested previously^3,12,17,18,61^, to the best of our knowledge, CA have never before been visualized within cell cytoplasm. Here, we convincingly show intracellular CA within endfoot processes of perivascular glial cells applying advanced image processing procedures for 3D reconstruction and volume rendering. CA proximity to blood vessels has been previously observed^3,12,18^, however no quantitative analyses have unambiguously demonstrated CA clustering in this area or visualized CA within the glymphatic system. In this study, we have performed quantitative analysis on 355 CA in combination with exhaustive 3D image analysis to prove that CA cluster towards the vascular system. Taken together, these results strongly suggest a link between CA and the glymphatic system and indicate CA origin within astrocytic feet.

The location of CA within the brain vascular system may corroborate the hypothesis that CA not only sequester products that are potentially hazardous to neurons, but also play a role in their removal from the brain^3,12^. We suggest that CA originate from aggregated cell components that form when intracellular biochemical properties are perturbed, and that the bulk of the membranous and glycosylated cellular material present in CA is of lysosomal origin. Lysosomes possesses a polysaccharide-based coating composed of highly glycosylated membrane proteins^62,63^. Hence, an abundance of lysosome-derived material within CA, may actually be responsible for the typical PAS staining used to identify them, rather than polysaccharide amyloid fibrils. Given the lack of visible amyloid fibrils and the presence of membrane fragments and lysosome-like vesicles, our 3D EM data strongly suggest that this is the case.

We postulate the following sequence of events: At some stage, the lysosomal degradation pathway may become insufficient, and waste material accumulates inside astroglia, which form a bridge between neurons and the glymphatic system. This material might originate from the astroglia themselves, from neurons and/or from blood brain barrier leakage. When the concentration in astroglia feet reaches a critical level, aggregation starts at one or more points, i.e., the growth of one or more CA is initiated to sequester the waste. Lipid-rich cellular clearance organelles accumulate at the agglutination points to digest it, locally changing the physical-chemical conditions and attracting other solutes, molecules and CA. Under normal physiological conditions, the small aggregates, i.e., the initial CA, are disposed of and cleared by the perivascular system of the brain, directly involving the glymphatic system. Under degenerative conditions the clearance rate slows down and the amount of material to be cleared increases. CA also increase in size by fusion and/or acquiring more waste material, ultimately disrupting the cell and becoming extracellular. As they are then surrounded by cell debris, extracellular CA continue to grow and remain at their origin site close to blood vessels.

Our hypothesis aligns with the protective role of CA^3,12^ and with new ideas that define cellular aging as a progressive disruption of phase-separated intracellular membrane-less compartments. These compartments can be envisaged as liquid droplets that are stable within the cytoplasm. They control spatiotemporal and diffusion-limited biochemical reactions and are extremely sensitive to changes in pH and protein concentration. When the balance is disrupted, they convert into structures with different physical and structural properties, such as hydrogels and aggregates^64,65^.

Perhaps the primary evolutionary role of all brain inclusion bodies was “protective”. However, under adverse conditions they become too abundant to be cleared and switch to a reservoir of cell death products, which leads to pathological phenotypes when neurons are affected. In the case of CA, there is no pathological phenotype, since CA do not directly affect neurons. Our results might explain why cellular and animal models have thus far failed to reproduce brain inclusion bodies appearance, and promise to inspire new studies that examine aging in the context of lipid metabolism^66^ and aggregation. Indeed, CA may be a key player that helps us to understand aging and degeneration.

To conclude, although we cannot yet explain CA growth and formation mechanism, our data radically change the model of their structure. The detection of fragmented lipid bilayers together with disrupted mitochondria and vesicle structures reminiscent of lysosomes and endosomes in CA by high-resolution 3D EM ultrastructural analysis, and the unexpected absence of ‘amyloid’ fibrils, is anticipated to incite novel hypotheses about CA, brain body formation, and the biological implications of their presence in aging and disease.

## Methods

### Human post-mortem brain tissue samples

Post-mortem tissue samples from PD and aged, non-demented donors (Supplementary Table S1), all with 5–8 hrs post-mortem delay (Supplementary Table S1), were obtained from the Netherlands Brain Bank (NBB, www.brainbank.nl), the Normal Aging Brain Collection (Dept. Anatomy and Neurosciences, VUmc, the Netherlands) and the Institute of Medical Genetics and Pathology of the University Hospital Basel, Switzerland.

All protocols of the Netherlands Brain Bank (NBB), Netherlands Institute for Neuroscience, Amsterdam (open access: www.brainbank.nl), and of the Normal Aging Brain Collection (NABC), VU University Medical Center, Amsterdam, were approved by the Medical Ethical Committee (METC), VU University Medical Center, Amsterdam, the Netherlands. For brain samples and/or bio samples obtained from the NBB, all material has been collected from donors for or from whom a written informed consent for a brain autopsy and the use of the material and clinical information for research purposes was obtained by the NBB. For brain samples obtained from NABC, all material has been collected from donors for or from whom a written informed consent for a autopsy and the use of the material and clinical information for teaching and research purposes was obtained by the department of Anatomy and Neurosciences, VUmc, the Netherlands.

For samples from the Institute of Medical Genetics and Pathology of the University Hospital Basel, the histological analysis of all brain specimens was authorized by the ethics committees on human studies of the cantons of Basel, Switzerland. Explicit consent was obtained for all cases. The guidelines of the ethics committee northwest and central Switzerland (www.eknz.ch) were strictly followed for the analysis of all human samples. Specimens were collected and archived as formalin-fixed paraffin blocks by the Institute for Medical Genetics and Pathology, University Hospital Basel.

The detailed neuropathological and clinical information about the samples was obtained in compliance with local ethical and legal guidelines.

In all cases, brain tissues were collected using a rapid autopsy protocol developed by the NBB at VUmc. At autopsy, four 0.5 cm-thick adjacent brain slices of the mesencephalon and hippocampus (mid) were collected. For EM, 1–2 mm^3^ cubes of the ventral part of the *Substantia nigra*, *pars compacta* (SNpc), *canalis centralis* and hippocampal region (CA2) were dissected and fixed for 6 hours in a mixture of 2% paraformaldehyde/2.5% glutaraldehyde in 0.15 M cacodylate buffer with 2 mM calcium chloride, pH 7.4 and then washed with PBS.

Neuropathological assessment was performed on formalin-fixed and paraffin-embedded sections (6 μm thick), collected from multiple brain regions according to the guidelines of BrainNet Europe. Briefly, 6 µm thick sections were cut and stained used H&E, Congo red, Gallyas silver stain, and immunohistochemistry against amyloid-β (clone 6 f/3d, 1:100, DAKO, United States), hyperphosphorylated tau (clone AT8, 1:100, Innogenetics, Belgium) and α-synuclein (clone KM51, 1:500, Monosan, The Netherlands). For pathological staging of amyloid-β, neurofibrillary and neuritic plaque and α-synuclein, pathology, diagnostic criteria were used according to the BrainNet Europe^67–69^ and the National Institute on Aging-Alzheimer’s Association guideline^70–73^.

### Histological staining analysis

8 μm-thick paraffin-embedded post-mortem human brain tissue samples from all donors were deparaffinized and stain following the H&E protocol^73^. 6 μm-thick paraffin-embedded post-mortem human brain tissue sections were deparaffinized and processed for PAS staining^73^.

### Serial block-face SEM imaging

Post-mortem human brain tissue was fixed in 2% filtered paraformaldehyde (Electron Microscopy Sciences, EMS)/2.5% glutaraldehyde (EMS) in 0.15 M cacodylate buffer supplemented with 2 mM calcium chloride, pH 7.4, washed in 0.15 M cacodylate buffer with 2 mM calcium chloride and kept at 4 °C for 1–2 days. 60 μm thick slices were then cut and collected at RT using a vibratome, washed in cold 0.15 M cacodylate buffer with 2 mM calcium chloride. The human brain slices were immersed in freshly prepared 2% KFeCN in 0.3 M cacodylate buffer with 4 mM calcium chloride and 4% OsO_4_, pH 7.4 for 1 h on ice, washed 5 times for 3 min with double distilled H_2_O (ddH_2_O) at room temperature (RT) and incubated in 0.22 μm-filtered thiocarbohydrazide (TCH) solution for 20 min at RT. Afterwards, they were washed 5 times for 3 min in ddH_2_O and immersed in 2% OsO_4_ in ddH2O at RT for 30 min, washed 5 times for 3 min in ddH_2_O and placed in 1% uranyl acetate in ddH_2_O at 4 °C overnight. The slices were then washed 5 times for 3 min with ddH_2_O at RT, immersed in lead aspartate solution at 60 °C for 30 min, washed 5 times for 3 min with ddH_2_O at RT and dehydrated in ice cold EtOH 6 times for 5 min at EtOH concentrations of 25%, 50%, 70%, 90%, 100%, and 100%. Next, they were immersed in 50% Durcupan (Sigma Aldrich):ethanol for 30 min, then in 100% Durcupan for 1 h, and incubated in 100% Durcupan overnight all at RT. Following this, the slices were immersed in freshly-prepared 100% Durcupan for 2 h and finally embedded in Durcupan at 60 °C over a time period of 48 h^74,75^. After the resin had hardened, 1 mm^2^ areas of the 60 μm-thick, resin-embedded tissue slices were cut at RT using a razor blade, mounted on standard aluminum pins, sputter-coated with gold and platinum in a vacuum system to enhance conductivity for SEM, and directly transferred to the chamber of a SEM for imaging. An FEI Quanta 200 FEG (Thermo Fisher, USA) equipped with a physical microtome (3View, Gatan, Pleasanton, CA, USA) mounted directly inside the microscope’s observation chamber was employed. An accelerating voltage of 5 keV, a spot size of 3, a scanning speed of 2 µsec/pixel, and high or low vacuum mode depending on the conductivity of the sample, were used for the serial block-face (SBF) imaging. After removal of an ultrathin (70 nm thick) layer using the 3View system, an image of the surface of the remaining specimen block was acquired using the Digital Micrograph software (Gatan), followed by further iterative section removal and SEM imaging. Such serial surface images were collected at 7–16 nm/pixel resolution in both the X and Y axes for specific ROIs, i.e., regions of the 1 mm^2^ tissue surface displaying a CA; ROIs were selected so that the CA was roughly centered. Alternatively, the whole 1 mm^2^ tissue block surface was scanned using a pixel size of 80 nm and 70 nm-thick layers were removed from the block until the entire 60 μm thick, resin-embedded tissue slice had been imaged. In both cases, the recorded SBF-SEM image stacks were digitally aligned, and reconstructed into 3D z-stacks/tomograms using the TrakEM2 module of *Fiji* (NIH ImageJ)^76^. 3D tomograms of tissue, representing roughly 15–50 µm-wide areas in the Z dimension were collected. These were large enough to contain entire CA.

### Correlative TEM and transmission electron tomography

For this work, half of a CA was imaged by SBF-SEM as detailed above, and the remaining portion by TEM. SBF-SEM imaging of a ROI was interrupted when the quality of the images and sample conductivity were good, when the CA was positioned towards the center of the ROI and when there was sufficient tissue left in the resin block. The remaining specimen block was removed from the SEM chamber, and individual 50 nm-thick sections were cut at RT using a physical ultramicrotome (Ultracut EM UC7; Leica Microsystems, Germany). Sections were collected on TEM nickel grids covered with a carbon-stabilized Formvar film (EMS) and used for correlative TEM imaging.

For TEM data acquisition, samples were imaged at RT in a Talos Arctica TEM (FEI, Thermo Fisher Scientific, USA) operated at 200 kV, a T12 (FEI, Thermo Fisher Scientific, USA) operated at 120 kV and a Philips CM10 (FEI, Thermo Fisher Scientific, USA) operated at 80 kV. Electron micrographs were recorded on a 4096 × 4096-pixel CMOS camera (FEI Ceta, Thermo Fisher Scientific, USA) and VELETA (EMSIS GmbH, Germany) camera.

For 3D TEM tomography, samples were imaged at RT in a Titan Krios (FEI, Thermo Fisher Scientific, USA) operated at 300 kV acceleration voltage, using a Quantum-LS energy filter (20 eV zero loss filter) and a K2 Summit direct electron detector (Gatan, Pleasanton, CA, USA) positioned after the energy filter. Tilt series were recorded using the SerialEM software^77^ according to the ‘dose-symmetric Hagen tilt-scheme’, which begins at low tilt and then alternates between increasingly positive and negative tilts to maximize the amount of high-resolution information maintained in the tomogram^78^. Images for the tilt series were collected at 3° increments from −60° to +60° at a nominal defocus ≤1 µm. The final pixel size was 4.311 Å at the specimen level.

Tilt series alignment by cross-correlation and patch-tracking followed by 3D reconstruction of unbinned tomograms were performed using *etomo* of the IMOD software^79^. The resulting tomograms were binned by a factor of 4 in all dimensions using IMOD. Anisotropic filtering and semi-automatic 3D color segmentation of the tomograms was performed by user-interactive thresholding and volume rendering using the Amira 6.0 software (FEI). Movies were generated using the Amira 6.0 software (FEI).

### Local texture analysis

Sub-tomographic texture analysis was performed using the *Dynamo* software^80–82^.

To analyze the texture of a given 3D area inside a tomogram, the boundaries of the area were manually identified. Afterwards, high contrast locations were selected and used as centers for the extraction of cubic volumes of 30 × 30 × 30 pixels (i.e., 51.7 nm side length), yielding sets of ~100 cubic particles per area. Mutual alignment of particles extracted from the same area was performed through 20 iterations of sub-tomogram averaging, measuring until convergence, i.e., until no further improvement of the alignment parameters was detected by additional iterations.

### Clustering analysis

To test the hypothesis that CA are more often located in close proximity to blood vessels in the ROIs (areas of hippocampus and SN close to the ventricle), three entire 1 mm × 1 mm × 60 μm resin-embedded tissue slices were examined by SBF-SEM by repeatedly imaging the 1 mm^2^ surface of the block (pixel size of 80 nm) and removing 70 nm-thick layers, as described above.

The most suitable block was analyzed further. All blood vessels and CA present in the volume represented by the image stack were traced and reconstructed using the *Dynamo* software. For the statistical analysis, the distance between a CA and a blood vessel was defined as the minimum distance between the CA and the surface of the blood vessel, abstracted as a triangulated mesh. The distance between each CA and its closest blood vessel in the 3D space was computed by measuring the distance between the CA and every blood vessel present and selecting the closest one. The size of the blood vessel was irrelevant.

To analyze the distance profile obtained, a computer simulation was performed to determine the distance distribution expected for a Poisson distribution of the CA, i.e., if the CA were located at independent random positions relative to each other and the blood vessels. The simulation was computed for ten thousand randomization sets. Every randomization set contained the same CA as the experimental data set, but the location of each was randomized according to a Poisson distribution. For each randomization, the distance profile of the randomized CA relative to the blood vessels was computed, allowing the expectation and variance of the number of CA below a given distance to their closest blood vessel to be calculated.

### Quantification and statistical analysis

CA was manually quantified in histological human brain sections from all donors stained by PAS and H&E method in *Fiji* (NIH ImageJ) by multi-point tool, and areas were measured by polygon selection tool. The concentration of CA per mm^2^ was calculated and graphically represented using GraphPad PRISM software.

Shape and size of CA in the 3D EM data set were computed in a semiautomatic fashion: The approximate location of each CA was selected by visual inspection. These estimates were used to feed an image processing algorithm based on texture and edge detection that determined the final values for radiuses and heights reported on Supplementary Fig. S5a. Mean values and SD of CA diameters were statistically assessed using GraphPad PRISM as well as a description of the CA population in the 3D EM data set reported in Supplementary Fig. S5b,c.

## Electronic supplementary material

Supplementary Information

Movie 1

Movie 2

Movie 3

Movie 4

Movie 5
