# Supplementary material for: Cerebral Corpora amylacea are dense membranous labyrinths containing structurally preserved cell organelles
Source: Sci Rep. 2018 Dec 21;8:18046. doi: 10.1038/s41598-018-36223-4 (PMC6303404; doi:10.1038/s41598-018-36223-4)
Supplement: Supplementary file 1 — Supplementary Information [file 41598_2018_36223_MOESM1_ESM.pdf]

Supplementary info for

Cerebral *Corpora amylacea* are dense membranous labyrinths containing structurally preserved cell organelles

Authors

Paula P. Navarro[1], Christel Genoud[2], Daniel Castaño-Díez[1], Alexandra Graff-Meyer[2], Amanda J. Lewis[1], Yvonne de Gier[3], Matthias E. Lauer[4], Markus Britschgi[5], Bernd Bohrmann[5], Stephan Frank[6], Jürgen Hench[6], Gabriel Schweighauser[6], Annemieke J.M. Rozemuller[7], Wilma D.J. van de Berg[3], Henning Stahlberg[1*], and Sarah H. Shahmoradian[1,8*]

[1] Center for Cellular Imaging and NanoAnalytics (C-CINA), Biozentrum, University of Basel, Basel, Switzerland

[2] Friedrich-Miescher Institute for Biomedical Research, Basel, Switzerland

[3] Department of Anatomy and Neurosciences, Section: Clinical Neuroanatomy, AO|2M, Amsterdam Neuroscience, VU University Medical Center, Amsterdam, The Netherlands

[4] Roche Pharma Research and Early Development, Chemical Biology, Roche Innovation Center Basel, Basel, Switzerland

[5] Roche Pharma Research and Early Development, NORD DTA/Neuroscience Discovery, Roche Innovation Center Basel, Basel, Switzerland

[6] Division of Neuropathology, Institute for Medical Genetics and Pathology, University Hospital Basel, Basel Switzerland

[7] Department of Pathology, Amsterdam Neuroscience, VU University Medical Center (VUmc), Amsterdam, The Netherlands

[8] Present address: OFLC/110, Paul Scherrer Institute, Villigen, Switzerland

* **Correspondence to:** Henning Stahlberg (henning.stahlberg@unibas.ch) and Sarah Shahmoradian (sarah.shahmoradian@psi.ch).

Supplementary Tables and Figures

Donor	Sex	Diagnosed as	Age at death (years)	Brain weight (g)	PMD (hrs:min)	Source	CA identified by LM	CA analyzed by 3D EM	Data shown in this study from hippocampus	Data shown in this study from brainstem
A	Male	PD	77	1240	5:15	NBB	3974	6+1*	Table S2; Fig. 2a; Fig.S1a-b; Fig. S4a, g, k, s; Fig. S3a, c; Fig. S6a; Fig. S8; Movie S2A	Fig. S1c-d; Movie S5
B	Female	PD	90	1335	4:45	NBB	6516	319+1*	Table S2, Fig. 1; Fig. 2d-f; Fig. 4; Fig. S1g-h; Fig. S3c; Fig. S4b, d, j, l-p, r, t-p. I; Fig. S7; Movie S1; Movie S2B; Movie S3	Table S2; Fig. 2g, j, k; Fig. S1g-h; Fig. S2b; Fig. S4f, h; Fig. S6a; Movie S2F
C	Male	Aged	84	1195	3:30	NBB	4188	7+1*	Table S2; Fig. 2b,c; Fig. 3; Fig. S1e-f; Movie S2E; Movie S3	Table S2; Fig. 2h, i, l; Fig. 5; Fig. 6; Fig. S1e-f; Fig. S4c; Fig. S6b; Fig. S9; Movie S2D; Movie S4
D	Male	Aged	92	1210	7:45	Normal aging brain collection, VUmc	1083	-	Fig. S1i-j	Fig. S1k-l
E	Male	Aged	76	1261	5:20	University Hospital, Basel	220	3	-	Fig. S4e, i, q; Movie S2E (*canalis centralis*)
F	Male	PD	84	1430	4:50	NBB	500	1*	-	Fig. S6f

Table S1. Information about the postmortem human brain sample donors.

PD: Parkinson's disease. NBB: Netherlands Brain Bank. *TEM data.

Membrane type	Width (nm)
Non-CA cellular membranes	5.4 ± 0.8
CA membranes	5.4 ± 0.9

Table S2. Membrane cross-sectional diameters determined from 2D TEM images.

The cross-sectional diameter of membrane fragments within 10 different CA found in post-mortem brain from donors A, B and D is compared to the cross-sectional diameter of other cellular membranes in corresponding TEM images. Number of measurements, n = 600 in both cases, total number of 2D TEM images analyzed = 211; p = 0.2349, R-square = 0.001178; no significant differences among means ($p < 0.05$). As the membrane planes will rarely have been oriented parallel to the incident electron beam, these values are an underestimate of the actual membrane diameter, which is best measured in a 3D reconstruction (see Supplementary Fig. S6 and Discussion).

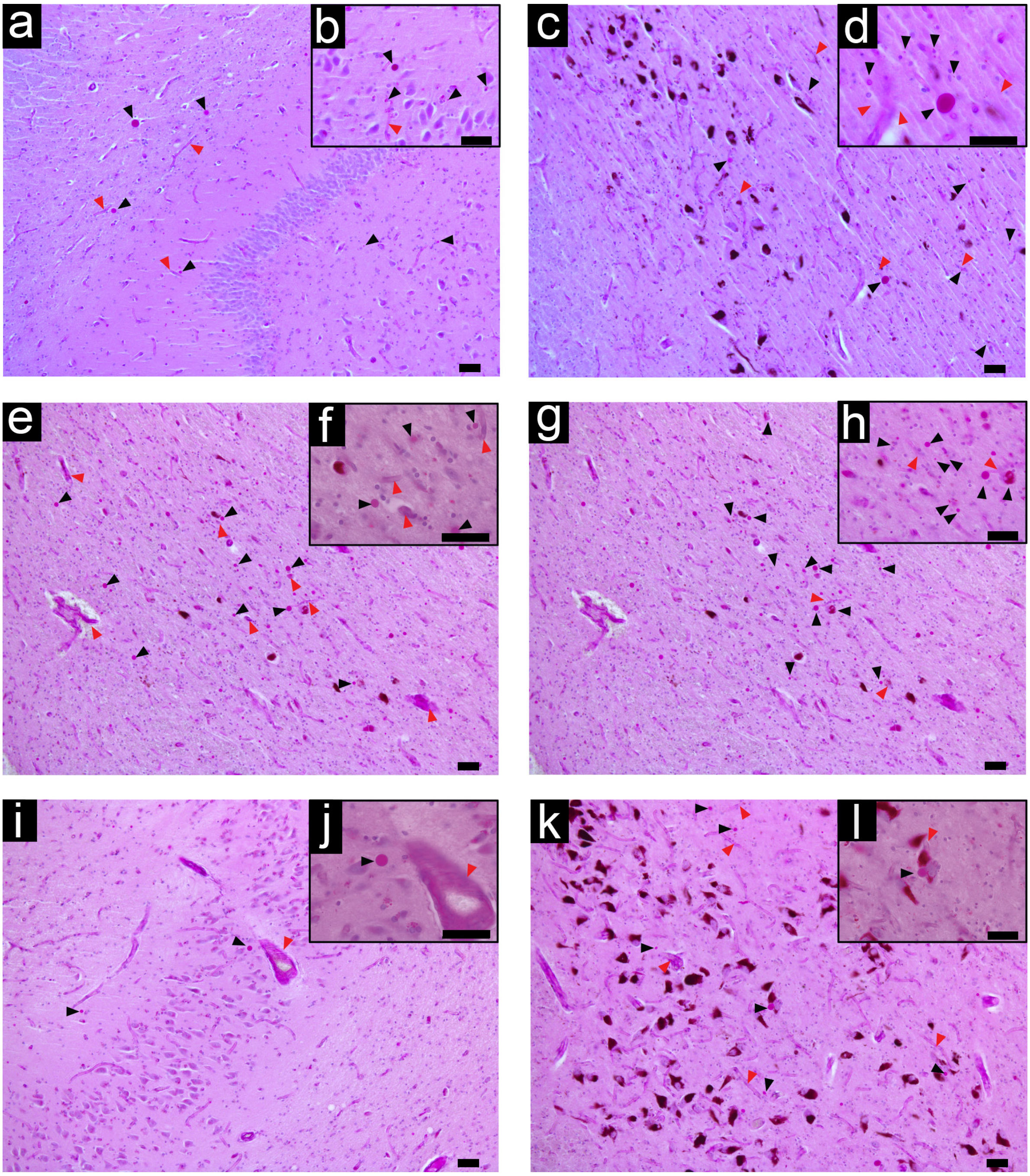

Supplementary Figure S1: *Corpora amylacea* identified by PAS. Sections were stained by PAS and imaged by LM. CA are visualized as purple/pink circular structures and small dots. (a) Donor A hippocampal section (towards ventricle). (b) Higher magnification image showing CA and blood vessels; some CA are close to cell nuclei and blood vessels. (c) Donor A brainstem SNpc. (d) Higher magnification image showing CA and blood vessels; some CA are close to cell nuclei and near blood vessels. (e) Donor C brainstem SNpc. (f) Higher magnification image showing CA, blood vessels and pigmented cells. (g) Donor B brainstem SNpc. (h) Higher magnification image showing CA and blood vessels; some CA are close to cell nuclei and cytoplasm as well as the blood vessels. (i) Donor D CA3 hippocampus. (j) Higher magnification image showing CA and blood vessels. (k) Donor D brainstem SNpc. (l) Higher magnification image showing CA near a blood vessels and within

pigmented cell. Black arrowheads = CA; red arrowheads = vascular vessels; white star = aged donors; other panels are PD donors. SNpc: *Substantia nigra pars compacta*. Scale bars = 50 µm.

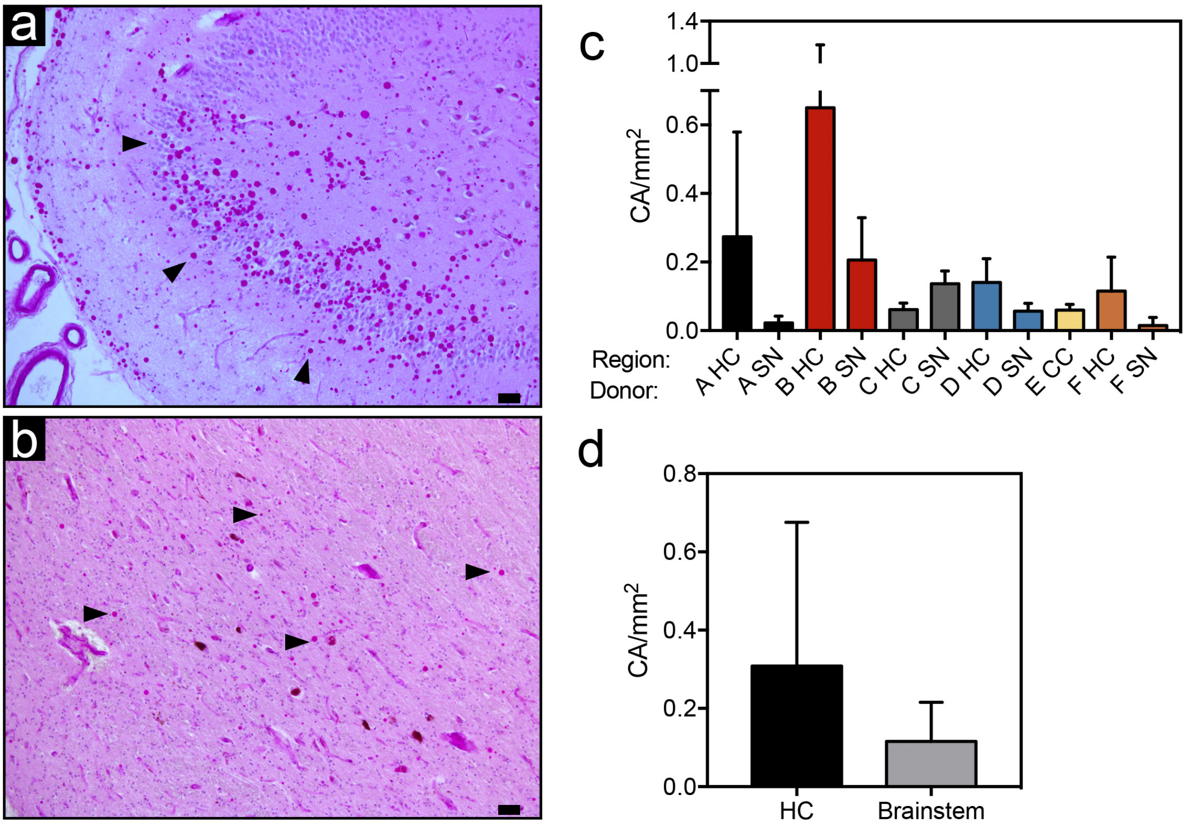

Supplementary Figure S2: Quantification of the concentration of *Corpora amylacea* identified by H&E and PAS staining in donors. (a) PAS stained section showing CA location and distribution in hippocampal dentate gyrus from donor B. (b) PAS stained section showing CA location and distribution in SN from donor C. (c) Sections imaged by LM were used to identified and quantify the number of CA/mm^2, per donor and per region. A HC: n = 8, mean = 0.27 ± 0.3; A SN: n = 3, mean = 0.02 ± 0.02; B HC: n = 6, mean = 0.65 ± 0.53; B SN: n = 6, mean = 0.2 ± 0.12; C HC: n = 4, 0.06 ± 0.2; C SN: n = 4, mean = 0.14 ± 0.04; D HC: n = 7, mean = 0.14 ± 0.07; D SN: n = 3, mean = 0.06 ± 0.02; E CC: n = 3, mean = 0.06 ± 0.02; F HC: n = 7, mean = 0.11 ± 0.1; F SN: n = 10, mean = 0.01 ± 0.02. (d) Number of CA per mm2 in the HC and brainstem region. HC, n = 24, mean = 0.3 ± 0.37; Brainstem: n = 19, mean = 0.12 ± 0.1; p-value = 0.034 by unpaired t-test. HC: hippocampus; SN: *Substantia nigra pars compacta*; CC: *Canalis centralis*. All data represented in this figure are shown as mean ± SD acquired from six donors. Scale bars = 50 µm.

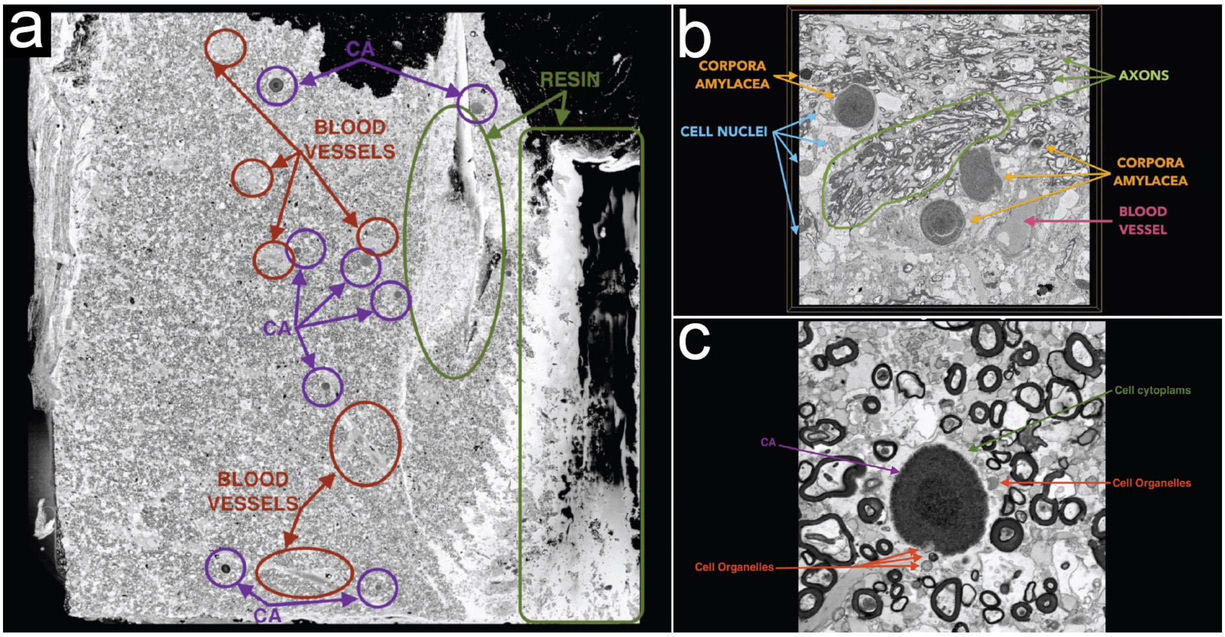

Supplementary Figure S3: Images describing *Corpora amylacea* and their cellular context in the human brain from Supplementary Movies 1 and 2. (a) Image from Supplementary Movie 1 showing CA and blood vessels within the resin-embedded tissue block. (b) Image from Supplementary Movie 2 indicating CA, axons, brain cell nuclei and blood vessels. (c) Image from Supplementary Movie 2 showing an intracellular CA and cell organelles at the edges of the CA.

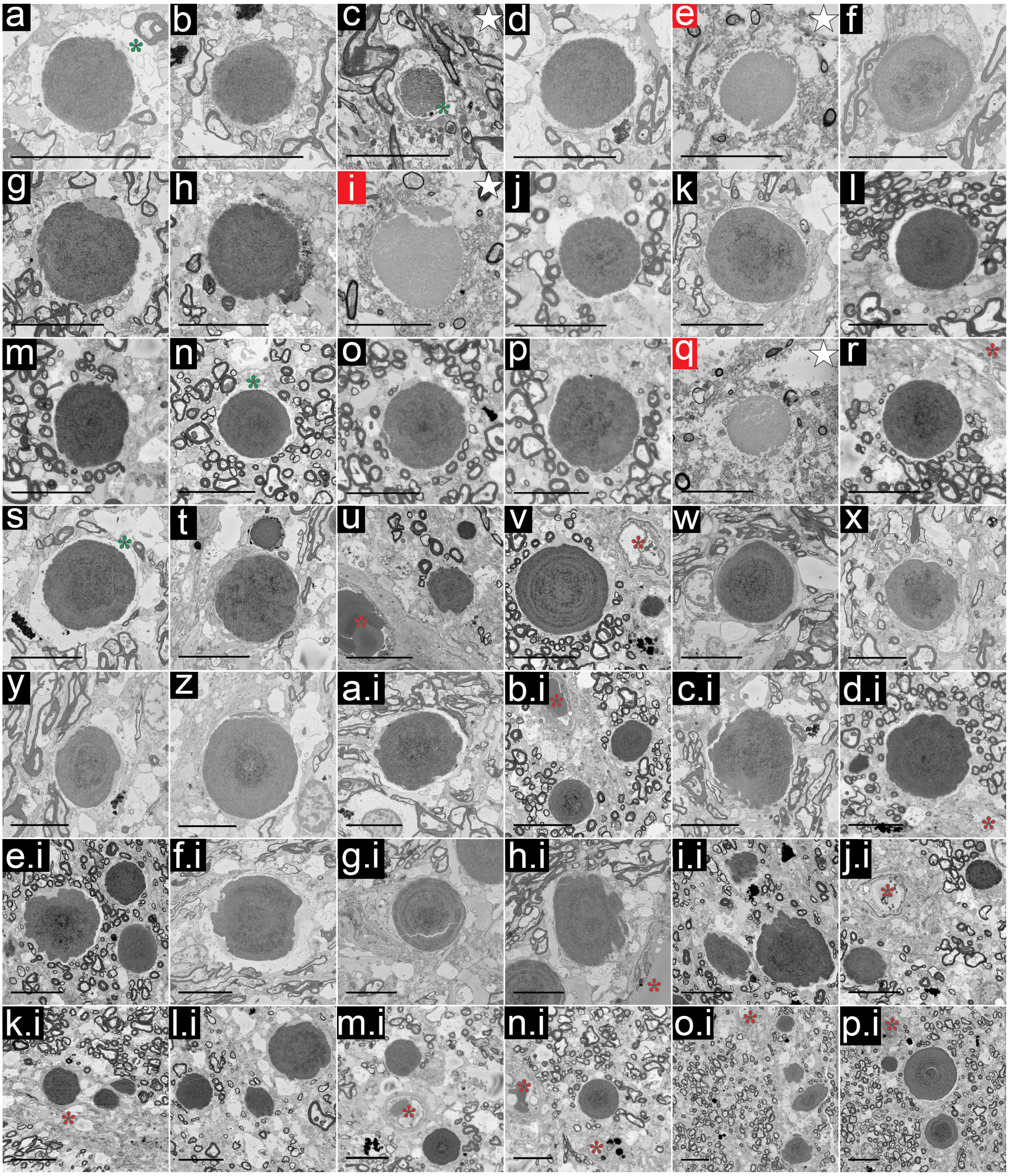

Supplementary Figure S4. Extensive gallery of heavy metal-stained *Corpora amylacea* as visualized by SBF-SEM. Each image represents a single central slice from a 3D stack. Green asterisk = CA surrounded by a membrane and thus considered to be intracellular; red asterisk = blood vessels. Red labels (e, i, q) = lower contrast images, because low vacuum conditions were used when acquiring the SBF-SEM data; white star = aged donors; other panels are PD donors. All data were acquired from hippocampal regions except (c) from (SNpc), and (e, i, q) from *canalis centralis*, which were from brainstem. SNpc: *Substantia nigra pars compacta*; SBF-SEM = serial block-face scanning electron microscopy. See Table S1 for details about the respective donors. Scale bars = 10 µm.

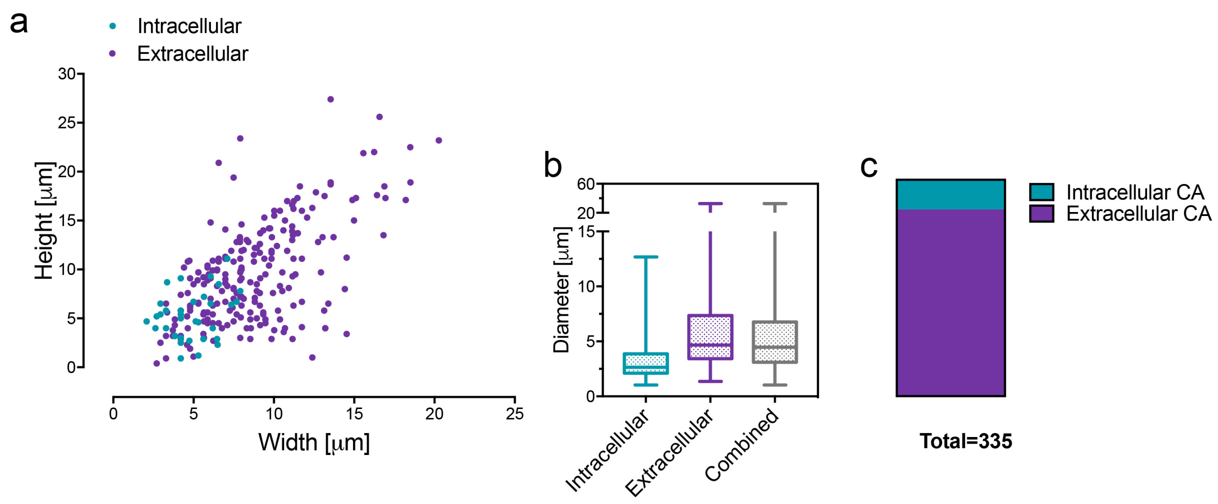

Supplementary Figure S5: Quantification of shape, diameter and number of intracellular and extracellular *Corpora amylacea* imaged by 3D EM. (a) For a total number of 258 3D full size CA width and height were measured in donors A, B, C and E. Each dot represents a CA, and color-coded as intracellular or extracellular CA. (b) For all 3D EM stacks containing CA in this study (n = 335) CA diameter was measured (µm) and represented as mean (horizontal color coded line in box) and diameter data distribution from donors A, B, C, and E. Intracellular: n = 46, mean = 3.58 ± 2.6; Extracellular: n = 289, mean = 5.99 ± 4.01, Combined: n = 335, mean = 5.66 ± 3.94. (c) Depiction of the intracellular (13.73%) and extracellular (86.27%) CA population as imaged in this study using 3D EM.

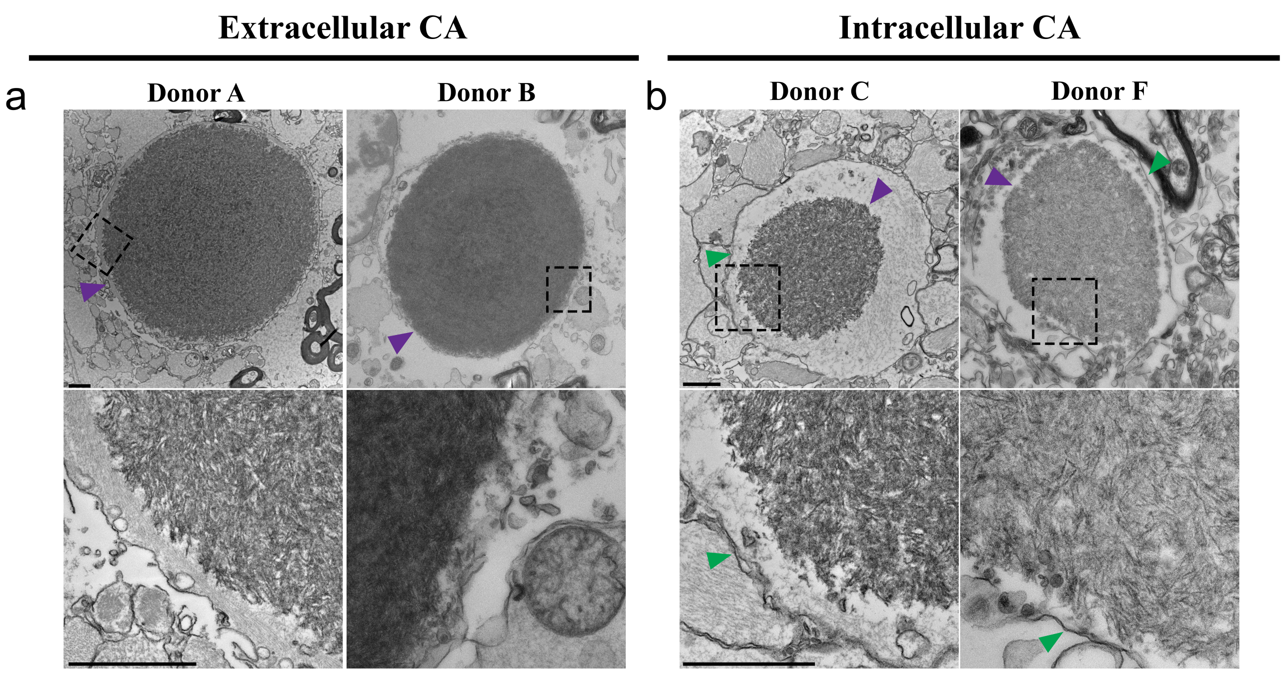

Supplementary Figure S6: TEM micrographs of intracellular and extracellular *Corpora amylacea* showing homogenous composition among different donors and brain regions. (a) Overview of extracellular CA in donor A hippocampus and donor B SNpc (top). High magnification images of boxed areas in top panel shown dense packed membranes composing the CA with vesicles, cytoskeletal protein filaments, cell organelles and neighboring cell extensions at their immediate surroundings (bottom). (b) Overview of intracellular CA in donor C hippocampus and donor F SNpc (top). High magnification images of boxed areas in top panel shown dense packed membranes composing CA, vesicle and cytoskeletal protein filaments being integrated into the CA, all enclosed by a cytoplasmic membrane (bottom). Purple arrowhead = CA; green arrowhead = cytoplasmic membrane. SNpc: *Substantia nigra pars compacta*. Dashed boxes represent the regions shown in the bottom panels. Scale bar = 1 µm.

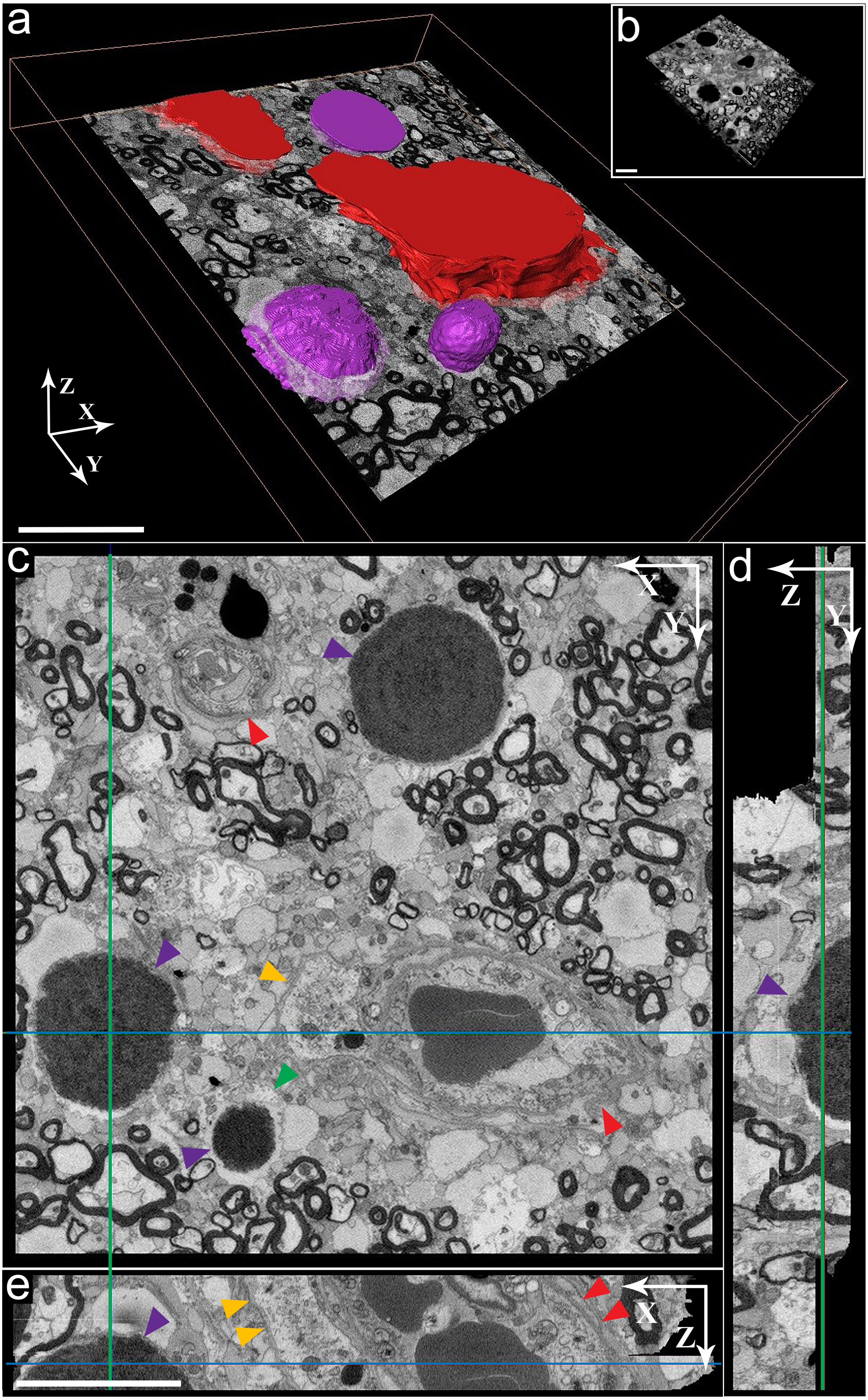
a
b
Z
X
Y
c
X
Y
d
Z
Y
e
X
Z

Supplementary Figure S7: 3D morphology and localization of *Corpora amylacea* in hippocampus of PD donor B, as visualized by SBF-SEM. (a) 3D surface reconstruction of the SBF-SEM data stack showing CA (purple surfaces) and blood vessel (red surfaces). (c-e) 3D visualization via 2D orthoslices of SBF-SEM data from CA found in. (c) XY orthoslice, (d) YZ orthoslice and (e) XZ orthoslice of the SBF-SEM stack displayed in (a and b). 2D image is shown in Figure 2f. Red arrowhead = blood vessel (basal lamina), purple arrowhead = CA, green arrowhead = cell containing CA forming a neurovascular unit, yellow arrowhead = pericyte forming a neurovascular unit. SBF-SEM = serial block-face scanning electron microscopy. Scale bars = 10 µm.

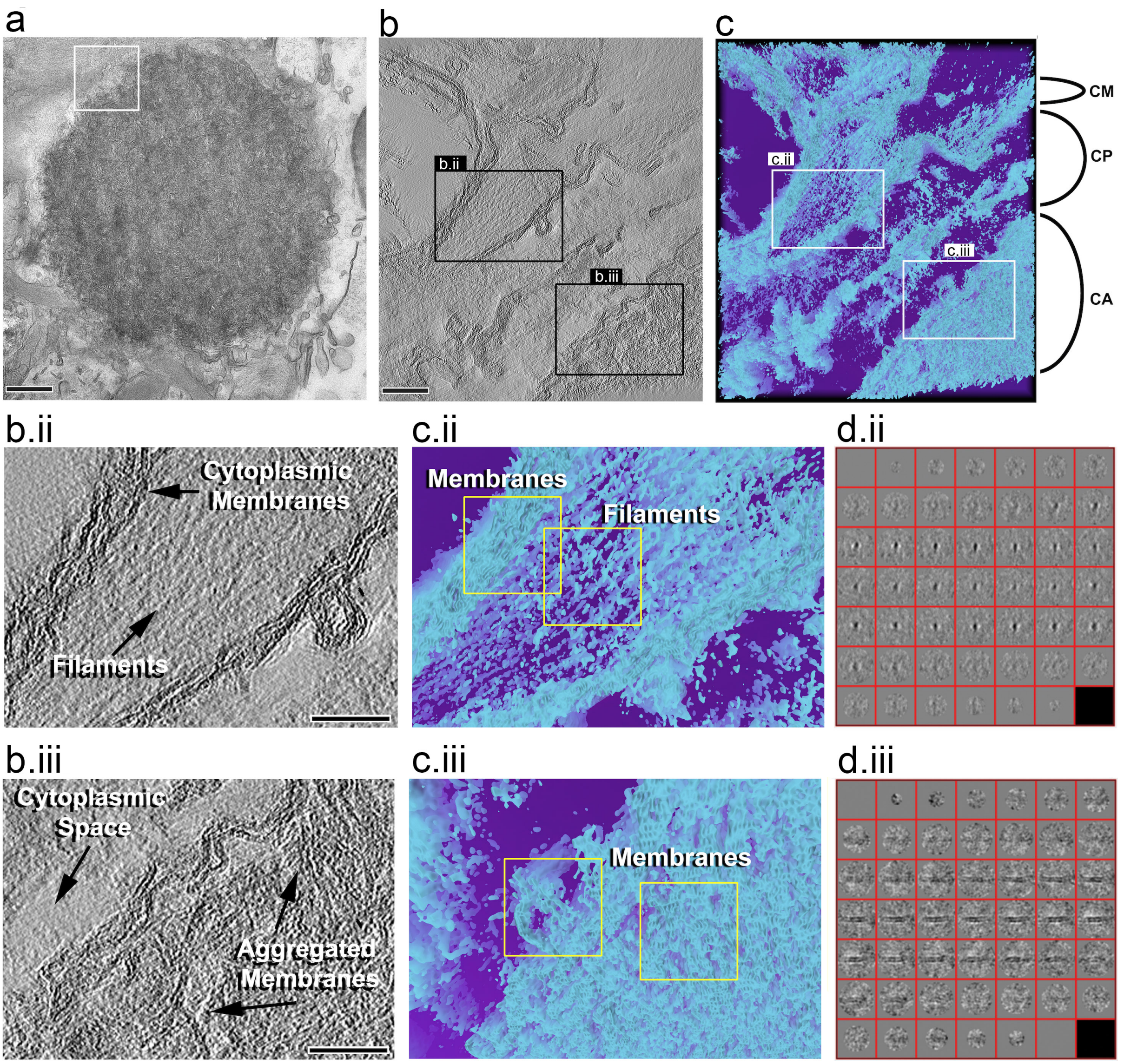

Supplementary Figure S8: Analysis of TEM tomograms of hippocampal *Corpora amylacea* reveals membrane structures in a PD donor. (a) TEM image of a 150nm-thick section of an extracellular CA in hippocampal CA2 of donor A. (b.i) Central slice of a tomogram of the periphery of the CA. The CA is in the bottom right corner. The region imaged is indicated by the white box in (a). (b.ii) Cellular structures adjacent to the CA in (b.i) (black box) show filamentous content. (b.iii) CA content includes membrane structures. (c.i) Color-segmented 3D display of tomograms corresponding to (b). CP = Cytoplasmic space, CM = Cytoplasmic membrane structures. (d.ii) Sub-tomogram averages of features from (b.ii) shows filamentous structure. (d.iii.) Sub-tomogram averages of CA content show a membrane signature. TEM = transmission electron microscopy. Scale bars = 1 µm in a; 50 nm in b.i, b.ii and b.iii. Width of Z-slices of the reconstructed subvolume averages in d.ii and d.iii is 105.6 nm.

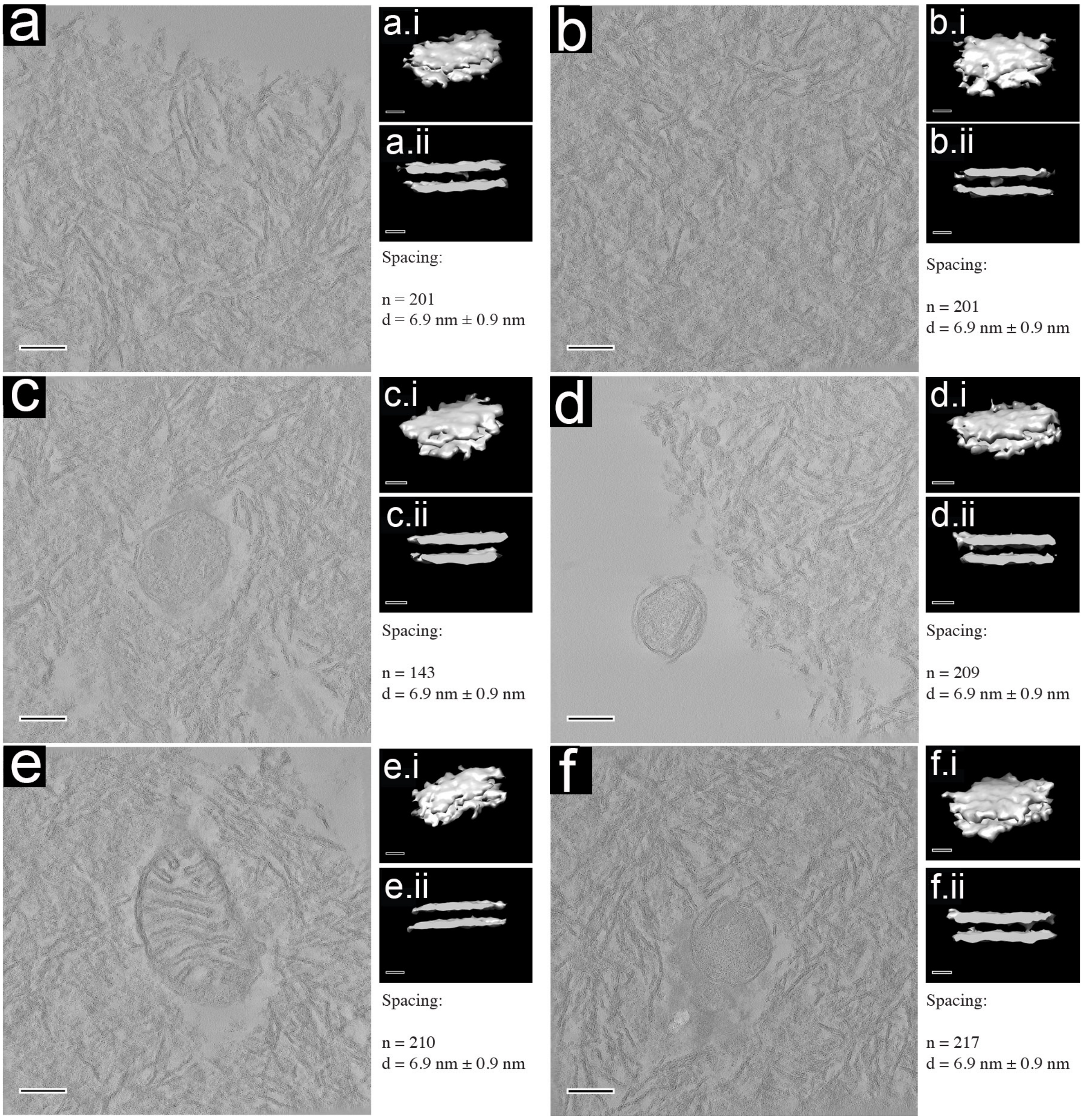

Supplementary Figure S9: Subtomogram averaging of *Corpora amylacea* electron tomograms reveals membrane structures in a non-demented aged donor. (a-f) Reconstructed tomogram projections of different regions of extracellular CA. Measured membrane diameters in subtomogram averages of 3D reconstructed patches of CAs are indicated in each panel. (a.i – f.i) top view and (a.ii – f.ii) side view of the average of the structures present in a – f, respectively. (a) The edge of the CA. (b) The core of the CA. (c) A core region of CA containing a vesicle. (d) Edge of the CA and a neighboring vesicle. (e) Core/edge region of the CA containing a mitochondrion. (f) Core region of the CA containing a vesicle. Panel (e) equates to Fig. 5a and panel (c) to Fig 5b. Scale bars = 200 nm in a - f ; 5 nm in a.i - f.i and a.ii - f.ii.

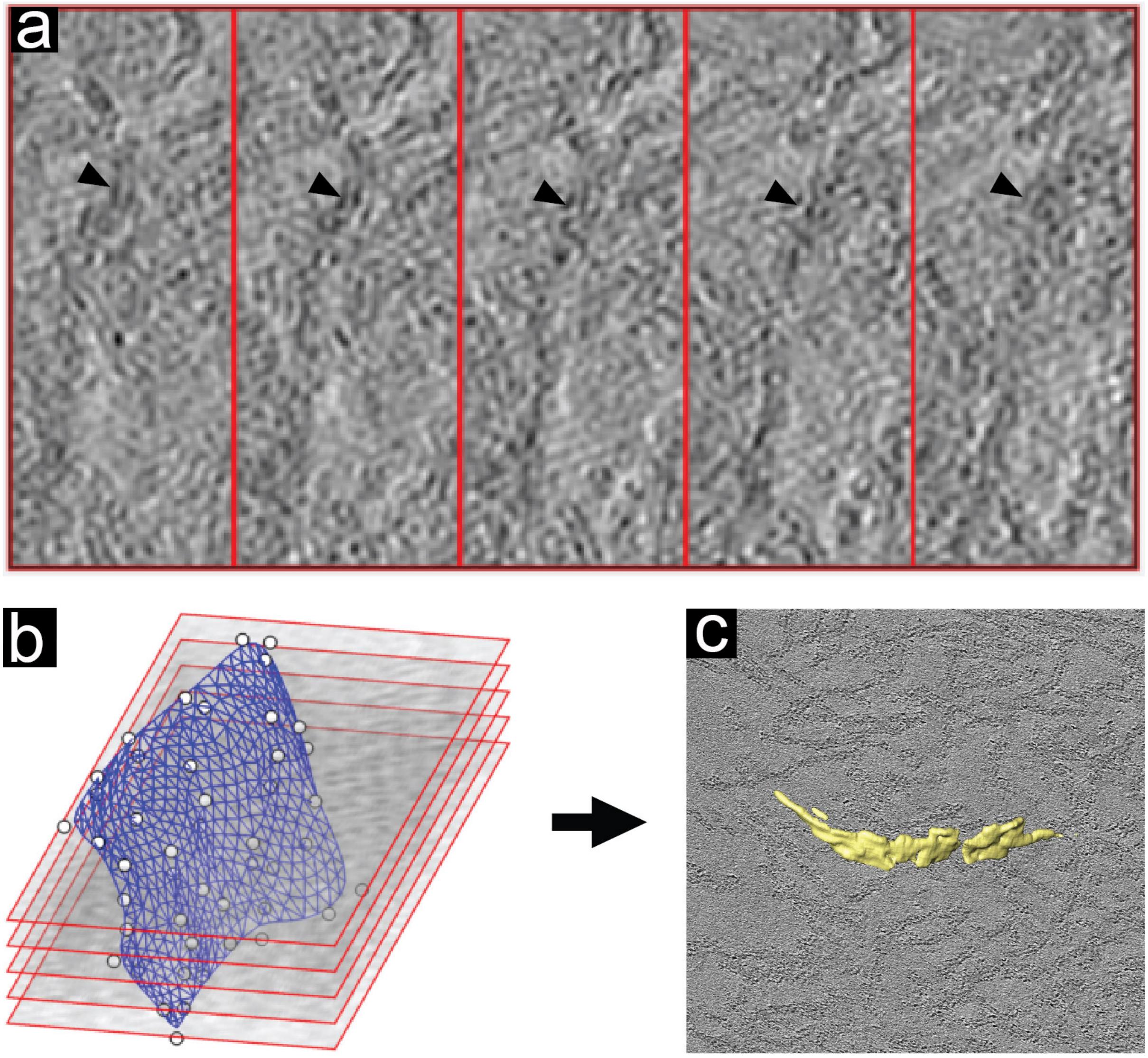

Supplementary Figure S10: Sub-tomogram averaging of membranes as found within the CA. (a) Series of Z-orthoslices of a TEM tomogram of CA, which show the membrane pattern and dynamics throughout the tomogram. (b) 3D triangulation of the membrane shown in (a) across the tomogram. (c) 3D surface reconstruction of a membrane (yellow volume) in the tomogram as generated by 3D-color segmentation. TEM = transmission electron microscopy.

Supplementary Movies

Supplementary Movie 1: SBF-SEM of the entire human brain tissue sample block. 3D reconstructed tomogram of a tissue sample block from the hippocampus brain region of donor B. Movie displays sample block shown in Figure 1c and statistically analyzed in Figure 4 and Supplementary Fig. S5. Time-lapse series were acquired with a rate of 4 fps in the compressed format JPEG. SBF-SEM = serial block-face scanning electron microscopy. Scale bar = 100 µm.

Supplementary Movie 2: SBF-SEM and 3D-color segmentation of *Corpora amylacea*. (A) 3D reconstructed tomogram of the tissue sample block from the hippocampus brain region of donor A. The different colors represent the different electron dense layers within the CA. Features such as cell organelles, CA, brain cells and blood vessels are indicated. (B). Extracellular and intracellular CA (second video) from donor B hippocampus. (C) Donor E brainstem *canalis centralis*. Data acquired at low vacuum. (D) Extracellular CA donor C, brainstem SNpc. This CA was partially imaged by SBF-SEM, and used for TEM / ET shown in Figure 5, 6 and Supplementary Figs. S9. (E) Intracellular CA from donor C hippocampal CA2 region. This stack is shown by 3D-surface reconstruction in Figure 3 and Supplementary Movie 3. 2D image is shown in Figure 2b. (F) Extracellular CA from donor B brainstem SN. 2D images of these CA are shown in Figure 2j and k. All 3D stacks shown were considered for CA shape and diameter measurements contained in Supplementary Fig. S5. Time-lapse series were acquired with a rate of 4 fps in the compressed format JPEG. Time-lapse series were acquired with a rate of 4 fps in the compressed format JPEG. SNpc: *Substantia nigra pars compacta*; SBF-SEM = serial block-face scanning electron microscopy. Scale bars: A = 10 µm; B, E, D and C = 5 µm; E = 3 µm.

Supplementary Movie 3: SBF-SEM of intracellular and extracellular *Corpora amylacea*. 3D reconstructed tomogram of tissue sample blocks from the hippocampus CA2 regions of donor B and C showing intracellular from Figure 3 and extracellular CA from Supplementary Figure S7 as well as the neurovascular unit forming the blood brain barrier (BBB) and vascular system of the brain. All 3D stacks shown were considered for CA shape and diameter measurements contained in Supplementary Fig. S5. Time-lapse series were acquired with a rate of 4 fps in the compressed format JPEG. SBF-SEM = serial block-face scanning electron microscopy Scale bars = 3 µm for donor C, and 8 µm for donor B, respectively.

Supplementary Movie 4: TEM tomograms of *Corpora amylacea*. 3D reconstructed, NAD-filtered, and 3D color segmentation of TEM tomograms depicting an extracellular CA in the brainstem SNpc of donor C. Movie displays tomograms that are analyzed in Figure 5, 6 and Supplementary Figure S9. Time-lapse series were acquired with a rate of 4 fps in the compressed format JPEG. SNpc: *Substantia nigra pars compacta*; TEM = transmission electron microscopy. Scale bar = 200 nm.

Supplementary Movie 5: TEM tomogram showing an overview of the edge of *Corpora amylacea* and the cellular environment. 3D reconstructed TEM tomogram of hippocampus CA2 from donor A showing the edge of and extracellular CA and the neighboring cellular environment. Movie displays tomograms analyzed in Figure S8. Time-lapse series were acquired with a rate of 4 fps in the compressed format JPEG. TEM = transmission electron microscopy.
